# Gene-environment interaction analysis of school quality and educational inequality

**DOI:** 10.1038/s41539-024-00225-x

**Published:** 2024-03-01

**Authors:** Kim Stienstra, Antonie Knigge, Ineke Maas

**Affiliations:** 1 Department of Sociology/ICS, Utrecht University, Utrecht, The Netherlands; 2 Department of Sociology, Vrije Universiteit Amsterdam, Amsterdam, The Netherlands

**Keywords:** Sociology, Education, Human behaviour

## Abstract

We study to what extent schools increase or decrease environmental and genetic influences on educational performance. Building on behavioral genetics literature on gene-environment interactions and sociological literature on the compensating and amplifying effects of schools on inequality, we investigate whether the role of genes and the shared environment is larger or smaller in higher-quality school environments. We apply twin models to Dutch administrative data on the educational performance of 18,384 same-sex and 11,050 opposite-sex twin pairs, enriched with data on the quality of primary schools. Our results show that school quality does not moderate genetic and shared-environmental influences on educational performance once the moderation by SES is considered. We find a gene-environment interplay for school SES: genetic variance decreases with increasing school SES. This school SES effect partly reflects parental SES influences. Yet, parental SES does not account for all the school SES moderation, suggesting that school-based processes play a role too.

## Introduction

Children who perform well in school are more likely to continue their education and obtain higher degrees, which in turn has numerous economic and social benefits including higher income, higher occupational status, and better health^1^. Inequalities in educational performance thus translate into inequalities in other domains. It is therefore important to know why some pupils perform better than others and how inequality can be reduced. An often studied source of differences in children’s performance levels are social differences among them, most notably, the influence of their family socioeconomic status (SES) background^2^. Another important source of differences are genetic differences between children^3^. The extent to which these family background influences and genetic influences play a role could be dependent on children’s school environment. Higher-quality schools could strengthen the influence of families and genes and thereby increase performance differences between pupils. Conversely, higher school quality could also decrease differences in performance because the family background and/or genetic influences are reduced in these schools.

There are opposing arguments on whether family background and genetic influences are multiplied or compensated in higher-quality schools. Social science literature suggests that family background influences could be stronger in high-quality schools, for example, because high-SES children benefit more from good learning opportunities as they enter school with better academic preparation^4^. Alternatively, the stable, stimulating, and resourceful learning environment in school could compensate for a less favorable home environment^5,6^. In that case, especially low-SES children may benefit from higher-quality schools. Similarly, the behavioral genetics literature provides opposing models on whether advantageous environments such as those provided by high-quality schools increase or decrease genetic influences. From the bioecological model, stronger genetic influences in higher-quality schools can be expected because the more resourceful and stable environment in such schools could promote the realization of genetic potential for greater achievement^7,8^. However, weaker genetic influences can also be expected. Following the diathesis-stress model^9^ and the idea of compensation interaction^10^, the absence of stressors and the more supportive learning environment in higher-quality schools could compensate for the realization of genetic risks for lower performance.

To what extent schools increase or decrease environmental and genetic influences on educational performance is important to know for reducing educational inequality. Whether schools reduce educational inequality does not only depend on the multiplicative or compensatory effect of schools. It also depends on which sources of differences in performance are seen as part of educational inequality. Family background differences in educational performance are commonly problematized and labeled as inequality of educational opportunity, or social inequality in education^11–13^. Concerning genetic differences, there are different standpoints on whether genetic differences (or genetic inequality) in educational performance are problematic and should therefore be reduced. On the one hand, genetic differences in educational performance can be seen as an indicator of opportunity for achievement^14,15^. If the realization of children’s innate talent is not restricted by social barriers, differences in educational performance would be to a larger extent explained by genes^14^. This (implicitly) assumes that differences due to good or bad luck in the genetic lottery are justified^16,17^. On the other hand, one cannot control their genetic endowment any more than their family background. Genetic differences could therefore, just as social differences, be interpreted as an unjust source of inequality^16,18,19^. Moreover, genetic differences in educational performance do not only reflect the realization of innate talent but also capture genetically influenced characteristics that negatively affect performance, such as behavioral and health problems^20^. If the genetic contribution to educational performance is larger in high-quality schools because such genetic risks are more expressed in these schools, most would agree that this is inconsistent with equality of opportunity.

Thus, family background differences are generally seen as unfair and as a form of educational inequality, and this source of inequality can be dependent on the school environment. The school environment may also affect the role of genetic differences in performance, but there are different perspectives on whether genetic differences are seen as an unfair and problematic source of inequality as well. We investigate how the school environment contributes to educational inequality and ask: ‘To what extent does the school environment increase or decrease genetic and family background influences on educational performance?’ We study this by using a twin design, which provides latent overall measures capturing genetic, shared (i.e., common, between-family) environmental, and non-shared (i.e., unique, within-family) environmental variance in educational performance^21^. We investigate how genetic and shared environmental variance varies across schools of different quality, also known as gene-environment interaction analysis.

While gene-environment interactions are predominantly focused on the family environment, more recently, interactions with the school environment have been studied. Prior twin studies yielded mixed results concerning whether more advantageous school environments increase or decrease genetic and shared environmental differences^22–25^. Concerning the interaction between schools and genetic influences in specific, recent studies also used polygenic indices (PGIs). PGIs are composite measures for each individual based on the correlation between genetic variants and an outcome, and therefore provide an estimate of an individual’s genetic liability to this outcome^26^. These studies investigated whether the association between educational attainment PGI and educational outcomes (including achievement, college completion, and dropping out of math, amongst others) differed between schools^27–29^. Results tend to support compensation, as those with a lower PGI benefitted more from schools with higher school-level achievement or SES.

While prior studies indicate that the school environment may moderate genetic and shared environmental influences on educational performance, it is unclear whether this can indeed be attributed to the school environment or reflect processes in the family environment instead. Children from high-SES parents more often attend high-quality schools^30,31^. School effects may therefore be confounded with family effects, and prior studies do not always take this sufficiently into account. Moreover, it remains an open question which specific school aspects play a role. In this study, we use a twin design to investigate whether the school environment moderates genetic and shared environmental influences while relying on many indicators measuring school quality. Additionally, we investigate to what extent the moderation by school quality is a moderation related to SES.

We study 29,434 same-sex and opposite-sex twin pairs (birth cohorts 1994–2007), that we identified in administrative data from Statistics Netherlands (*CBS*). These data cover the whole population and do not suffer from the self-selection bias that can affect twin samples^32,33^. Additionally, the number of observations provided by administrative data lead to ample power to detect genetic and shared environmental influences and their interactions with school quality. The administrative data contain children’s scores on a national standardized achievement test (*Cito* test) administered at the end of primary school around age 12. We enriched these data with many school quality indicators, as derived from the Dutch Inspectorate of Education.

The Netherlands provides an interesting context for investigating the role of school quality on educational inequality. The *Cito* test is a high-stakes test with major importance for children’s educational careers. Together with a recommendation of the teacher, the test result determines which secondary school track children will attend. Once enrolled in a particular track, opportunities to switch to a higher track are limited^34^. Educational inequality in this test score, whether it is related to social background differences and/or genetic differences, has thus large implications for future educational and career opportunities.

There are different theoretical arguments on how genetic and family background differences, and therewith educational inequality, depend on school quality. Although there is no clear definition of school quality, there seems to be a consensus that it entails at least two aspects: school resources and school culture. School resources refer to aspects that can (potentially) be bought either directly (e.g., educational materials) or more indirectly (e.g., educational time, teacher attention)^35,36^. School culture characteristics cannot readily be bought and are more difficult to change. These include norms, values, and expectations (e.g., academically oriented culture, high expectations), relationships (e.g., teacher-pupil relationships, cohesion), teaching and learning practices (e.g., structured instruction, differentiation), and larger organizational structures (e.g., educational leadership)^35,37^.

High-quality schools can be expected to both increase and decrease *genetic influences* on educational performance. An increase can be expected from the bioecological model^7,8^, according to which genetic potential for developmental outcomes such as greater educational achievement is more actualized with increased levels of proximal processes (i.e., enduring forms of interaction in the immediate environment, e.g., parent-child interactions). This model has generally been applied to the role of family environment in explaining cognitive ability, which has become known as the Scarr-Rowe hypothesis^38,39^. This hypothesis claims that in high-SES families, genetic potential is more fully expressed. Environments such as those provided by high-SES families can be seen as more advantaged and stable. They more often comprise different resources (e.g., material resources, cultural capital) and proximal processes that are more aligned with children’s genetic potential and are therefore expected to enhance genetic expression^8,40,41^. While the focus was originally on the realization of genetic potential for cognitive abilities, this has been extended to educational outcomes. For both outcomes, support for the Scarr-Rowe interaction has been mixed^40,42–44^. Support has been found mostly in the U.S., whereas in other western countries, no interaction or a reversed pattern has been found^42^. These mixed findings have been related to differences in socioeconomic inequality and welfare state arrangements, but also aspects of the study design including operationalizations and the size and representativeness of the sample^43,45,46^.

The bioecological model and Scarr-Rowe hypothesis could be applied to the impact of schools on genetic influences on educational performance. Similar to high-SES families, high-quality schools are more stable and resourceful environments. In these schools, higher levels of positive proximal processes (e.g., teacher-child interactions) can be expected, which implies that teachers’ behavioral patterns are more responsive to children’s characteristics and actions. This is seen as the principal mechanism through which genetic potential for effective developmental functioning is actualized^8^. Available school resources (e.g., more experienced teachers, more teacher attention) and school culture (e.g., monitoring students’ progress, differentiation) may make it easier to discover children’s specific talents. Additionally, a high-quality school environment is characterized by aspects that could lead to children developing their talents further, such as the availability of challenging materials and the presence of high-achievement norms.

Alternatively, from the diathesis-stress model, it can be derived that genetic influences decrease with increasing school quality. According to this model, the realization of genetic risk for lower performance (e.g., learning or behavioral problems) is more likely when there are more environmental risks and stressors^9,10^. The school environment in low-quality schools can be expected to have more environmental stressors, such as higher levels of classroom disorder and negative peer influences. Hence, the expression of genetic risks is more likely in such environments but decreases when such environmental stressors are less present as in high-quality schools. Moreover, high-quality schools have other positive features that may compensate for the realization of genetic risk^10^. For example, in high-quality schools, teachers may be more likely to notice specific risk factors for lower performance and these schools may also be better able to provide adequate support (e.g., remedial teaching).

Depending on the quality of the school, the influence of the *family environment* can also be expected to become either more or less important. In the sociological literature, several arguments are provided for why the influence of family background might be stronger in higher-quality schools. One is that children from high-SES families have a cumulative advantage and benefit more from a high-quality school environment because they enter school better academically prepared^4^. Based on the idea that ‘skills beget skills’, children’s skills gained early in life increase children’s capacity to benefit from later instruction in school^47,48^. For example, in high-SES families, children may develop more language skills because their parents tend to engage children more in conversations and use a richer vocabulary^41^. They may therefore understand instructional material better and reach higher performance levels in school. Another argument is that there is greater cultural correspondence between the home and school environment for high-SES children. The more ambitious and academically oriented culture in high-quality schools coincides with high-SES parents’ expectations and ambitions. For low-SES students, such a culture means a mismatch between their family and classroom experiences which may lead to negative self-perceptions and emotional distress, negatively affecting educational outcomes^49^.

Conversely, family background influences can also become less important in high-quality schools. According to the bioecological model, proximal processes do not only increase the realization of genetic potential, but also reduce, or buffer against, (shared) environmental differences in developmental outcomes^8^. Sociological literature provides more insight into how a higher quality school environment may reduce family background differences in performance. Children from disadvantaged families tend to grow up in a more unstable environment outside school, receive less parental support, and have access to fewer parental resources. The environment in high-quality schools may be especially important for them^5,6,50^. High-quality schools provide access to learning opportunities that overlap with those in socioeconomically advantaged families. If learning opportunities in families and schools substitute for each other, school resources typically benefit students from a less resourceful family environment more than they benefit high-SES students^4,51^. Also, school culture aspects (e.g., academic climate, good student-teacher relationships) improve student achievement, especially for children from more disadvantaged families^52^. These children could have a ‘differential sensitivity’ to such aspects in their school environment because they experience them less in their families^6^. Differences in educational performance attributable to family background may thus become less pronounced in high-quality schools.

When investigating how genetic and family background differences depend on school quality, it is important to consider socioeconomic selection into schools. Children from high-SES parents more often attend high-quality schools^30,31^. Consequently, higher-quality schools are not only characterized by their more advantageous resources and culture but also a high-SES composition. This composition may also affect educational performance. Moreover, the school’s SES composition may affect the influences of genes and the environment on educational performance. Partly, school SES influences overlap with those of school quality because they are correlated. Schools’ SES composition is associated with school characteristics such as teaching and instruction practices, and school organization and management processes^53^. For example, high-SES schools may more easily attract good and experienced teachers and have more rigorous curricula^54,55^. Since we explicitly measure school quality and rely on many indicators, we likely capture school-based influences related to school quality characteristics such as teaching and instruction practices, and school organization. However, school SES may also reflect other school-based mechanisms that are less captured by school quality, such as peer interactions^54^. For instance, high-SES students with higher aspirations, better study habits, and less disruptive classroom behavior may have a positive influence on the performance of other students^56^. Given such school-based mechanisms, some previous studies proposed that a large proportion of students from high-SES backgrounds is an indicator of school quality^57,58^. For this reason, it is worthwhile to study school SES in addition to school quality. Another important consequence of socioeconomic selection into school is that the school environment may capture family SES influences if these are not considered. High-SES parents tend to provide more stable and resourceful environments, just as high-quality schools do. Hence, parental SES may moderate genetic and shared environmental influences similar to school quality. Not considering this would overestimate the moderation by school quality.

Altogether, we explore whether school quality increases or decreases genetic and family background differences in educational performance. To provide more insight into the possible moderation of the school environment we also study if school SES increases or decreases genetic and family background differences in educational performance and explain part of the moderation effect of school quality. Lastly, we investigate if the moderation effect of schools is explained by parental SES.

## Results

### Genetic and environmental influences on educational performance

Before examining the *ACE-*moderation model, we first investigate unmoderated genetic and environmental influences on educational performance by decomposing the variance in educational performance in genetic (*A*), shared environmental (*C*), and non-shared environmental (*E*) variance. Since zygosity is unknown, we rely on comparing opposite-sex (OS) twins, characterized by an average genetic relatedness of 0.5, with same-sex twins from whom the exact genetic relatedness (i.e., *rSS*_*G*_) is uncertain. Therefore, we use different values of *rSS*_*G*_ (including 0.70, 0.75, and 0.80). The total variance in educational performance is ${V}_{{educ}}$ = 95.34. While the total amount of variance does not depend on *rSS*_*G*_, the variance decomposition differs. In our lower bound scenario (*rSS*_*G*_ = 0.70, Model 1, Table 1), genetic differences explain 90.9% of the variance in educational performance ($S{V}_{A}$ = 86.65 / 95.34 = 909) and we find no shared environmental variance. When we use *rSS*_*G*_ = 0.75 (Model 2, Table 1), we find that the variance in educational performance is to a lesser extent attributable to genetic differences (73.0%) and more to shared environmental variance (8.9%). When we further increase *rSS*_*G*_ (Model 3, Table 1), genetic variance becomes smaller and (non-)shared environmental variance larger. Altogether, 61–91% of the variance in educational performance can be attributed to genetic variance, 0–15% to shared environmental variance, and 9–24% to non-shared environmental variance.

**Table 1:** ACE model for cito for different values of rSS_G_ (N_SSpairs_ = 18,384, N_OSpairs_ = 11,050)

	Model 1 *(rSS*_*G*_ = 0.70)		Model 2 *(rSS*_*G*_ = 0.75)		Model 3 *(rSS*_*G*_ = 0.80)	
Parameter	Estimate	s.e.	Estimate	s.e.	Estimate	s.e.
Intercept	534.63^***^	(0.08)	534.63^***^	(0.08)	534.63^***^	(0.08)
*a*	9.31^***^	(0.06)	8.34^***^	(0.24)	7.62^***^	(0.22)
*c*	0.00	(0.04)	2.91^***^	(0.47)	3.78^***^	(0.32)
*e*	2.95^***^	(0.12)	4.16^***^	(0.16)	4.80^***^	(0.11)
*V*_*A*_	86.65^***^	(1.14)	69.61^***^	(3.96)	58.00^***^	(3.30)
*V*_*C*_	0.00	(0.00)	8.46^**^	(2.74)	14.26^***^	(2.42)
*V*_*E*_	8.69^***^	(0.71)	17.28^***^	(1.32)	23.08^***^	(1.02)
Freely estimated parameters	6		6		6	
Loglikelihood	−190,307.99		−190,307.98		−190,307.98	
Scaling correction factor	1.08		1.30		1.30	
AIC	380,627.98		380,627.97		380,627.97	

**p < 0.01, ***p < 0.001 (two-tailed test). Controlled for sex and year of birth. Robust standard errors accounting for clustering at the school level are shown in parentheses. Parameters *a*, *c*, and *e* refer to the unmoderated path coefficients capturing genetic, shared-environmental, and non-shared environmental influences, respectively. Squaring these path coefficients gives the variance components *V*_*A*_, *V*_*C*_, and *V*_*E*_.

Next, we sequentially include the main effects of school quality, school SES, and parental SES on educational performance (Supplementary Table 1). The estimated sizes of these associations do not depend on *rSS*_*G*_. School quality is statistically significantly associated with educational performance (*b* = 0.61, *β* = 0.06, *p* < 0.001). This is not substantial; each standard deviation (S.D.) increase in school quality is associated with a 0.61 point (0.06 S.D.) increase in the *Cito*-score. Additionally, sequentially including school SES and parental SES shows that parental SES has a much stronger association with educational performance (*b* = 2.89, *β* = 0.30, *p* < 0.001) and explains part of the association between educational performance and school quality and school SES. The school quality association with performance reduces to 0.24 (*β* = 0.02, *p* < 0.001). There is a relatively weak positive association between school SES and educational performance (*b* = 0.92, *β* = 0.09, *p* < 0.001) once we control for parental SES (and school quality). If there is shared environmental variance present, as is the case for *rSS*_*G*_ = 0.75 and *rSS*_*G*_ = 0.80, this is (almost) entirely explained by school quality, school SES, and parental SES.

### Genetic and environmental influences moderated by school quality and school SES

**Fig. 1:**
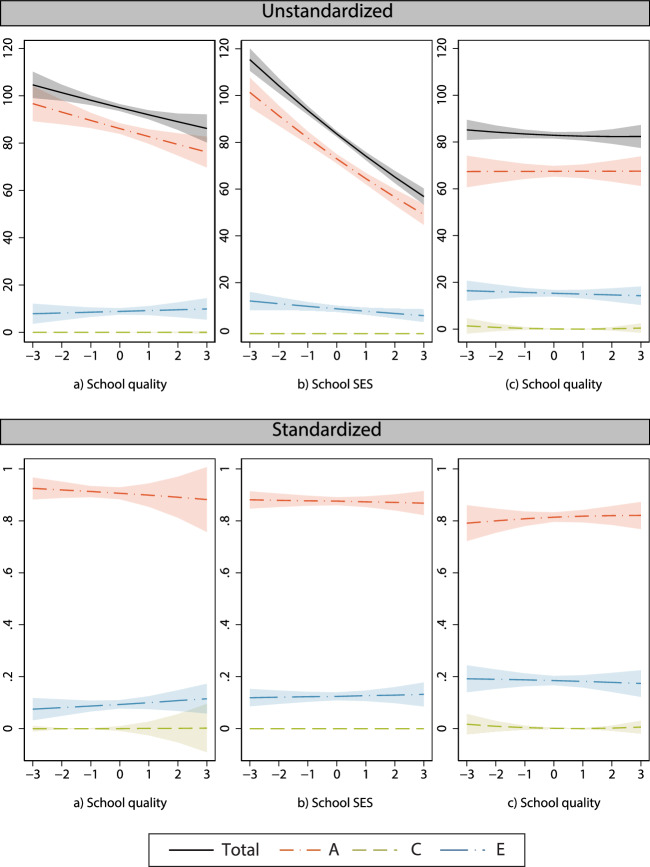
Unstandardized (top row) and standardized (bottom row) ACE moderations. Genetic (*A*), shared environmental (*C*), and non-shared environmental (*E*) variances of educational performance moderated by (**a**) school quality only, (**b**) school SES only, and (**c**) school quality controlled for the moderation of school SES and parental SES. Including 95% CI. Based on a model using a genetic correlation of *rSS*_*G*_ = 0.70.

Next, we test how school quality moderates genetic and environmental variance while using a genetic correlation of *rSS*_*G*_ = 0.70 (Model 1, Table 2). We could have used 0.75 and 0.80 as well, which we do in the next section as robustness checks. We find that with increasing school quality, genetic influence decreases statistically significantly, ${b}_{a}{SQ}$ = −0.019, *p* = 0.009 (Fig. 1a and Table 2, Model 1). Shared environmental variance is absent when using *rSS*_*G*_ = 0.70 and thus does not depend on school quality. Because of the decreasing genetic variance, the total variance in educational performance also decreases with increasing school quality. When this is considered by standardizing the *ACE* components, we see that the relative genetic influences barely decrease with increasing school quality (Fig. 1a).

**Table 2:** ACE model for cito with interaction effects of school quality (SQ) and school SES (N_SSpairs_ = 18,384, N_OSpairs_ = 11,050)

Parameter	Model 1		Model 2		Model 3		Model 4	
	(SQ only)		(School SES only)		(SQ and school SES)		(SQ, school SES, and parental SES)	
	Estimate	s.e.	Estimate	s.e.	Estimate	s.e.	Estimate	s.e.
Intercept	534.63^***^	(0.08)	534.65^***^	(0.07)	534.65^***^	(0.07)	534.67^***^	(0.06)
*a*	9.28^***^	(0.08)	8.90^***^	(0.06)	8.88^***^	(0.07)	8.22^***^	(0.07)
${b}_{a}{SQ}$	−0.19^**^	(0.07)			−0.05	(0.06)	0.00	(0.06)
${b}_{a}{SchoolSES}$			−0.52^***^	(0.05)	−0.51^***^	(0.05)	−0.31^***^	(0.06)
${b}_{a}{ParentalSES}$							−0.62^***^	(0.06)
*c*	0.16	(1.31)	0.00	(0.00)	0.13	(0.25)	0.26	(0.19)
${b}_{c}{SQ}$	0.10	(1.05)			0.39	(0.50)	−0.32	(0.24)
${b}_{c}{SchoolSES}$			0.00	(0.00)	-0.12	(0.16)	−0.84^***^	(0.23)
${b}_{c}{ParentalSES}$							0.71^***^	(0.19)
*e*	2.98^***^	(0.13)	3.35^***^	(0.10)	3.37^***^	(0.11)	3.91^***^	(0.09)
${b}_{e}{SQ}$	0.06	(0.13)			0.01	(0.10)	−0.04	(0.09)
${b}_{e}{SchoolSES}$			−0.17^*^	(0.08)	−0.17^*^	(0.08)	−0.05	(0.09)
${b}_{e}{ParentalSES}$							−0.22^**^	(0.08)
School quality	0.61	(0.07)			0.24^***^	(0.06)	0.22^***^	(0.06)
School SES			2.23	(0.06)	2.20^***^	(0.06)	0.91^***^	(0.06)
Parental SES							2.94^***^	(0.05)
Freely estimated parameters	13		10		17		21	
Loglikelihood	−217,429.91		−174,629.60		−216,384.47		−214,779.29	
Scaling correction factor	2.23		0.98		1.90		1.77	
AIC	434,885.82		349,279.20		432,802.93		429,600.58	

*p < 0.05, **p < 0.01, ***p < 0.001 (two-tailed test). A genetic correlation of *rSS*_*G*_ = 0.70 is used. Controlled for sex and year of birth. All continuous independent variables are *z*-standardized prior to the analyses. Robust standard errors accounting for clustering at the school level are shown in parentheses. Parameters *a*, *c*, and *e* refer to unmoderated path coefficients capturing genetic, shared-environmental, and non-shared environmental influences, respectively. The *b* coefficients refer to the moderation effects of *a, c*, and *e*, by school quality (SQ), school SES, and parental SES

Similar to our findings for school quality, our results show that school SES decreases genetic influence and does not affect shared environmental influence on educational performance (Fig. 1b and Table 2, Model 2). Although we did not have expectations on the moderation of non-shared environmental variance, we find that it statistically significantly decreases with increasing school SES (Model 2, Table 2). When the decreasing total variance with increasing school SES is taken into account, we do not find any moderations in the relative contribution of genetic and environmental variances (Fig. 1b).

### Simultaneous test of the moderating role of school quality and SES

The average SES of children in a school may explain part of the moderation effect of school quality. When we test the moderation by school quality and school SES simultaneously in Model 3 (Table 2), the model fits the data better than the model that only includes the moderation by school quality. When the moderation effects of school SES are taken into account, the genetic moderation by school quality is indeed reduced and no longer statistically significant. School SES could capture both school effects (e.g., peer group processes) and family effects. Therefore, we additionally include parental SES as a moderator in Model 4, which fits the data better than Model 3. This final model shows that the moderating role of school SES is partially attributable to parental SES. The previously found moderation of genetic variance by school quality is thus partly related to the selection of high-SES children in high-SES schools. When this is considered, we find no evidence for moderation effects of school quality anymore (Fig. 1c).

The final model shows that the moderating role of school SES is partially attributable to parental SES, but not entirely. School-based processes likely play a role too, as the genetic moderation effect by school SES becomes smaller in magnitude (a reduction of 40%) but a substantial part remains and is statistically significant (see Table 2, Model 4). We also find a statistically significant moderation of shared environmental variance by school SES once parental SES is controlled for. However, we find the evidence for decreasing shared environmental variance rather weak given the small amount of shared environment variance that is present to begin with. Lastly, the decreasing non-shared environmental variance by school SES that we found in Model 3 (Table 2), appears to be attributable to parental SES (see Model 4, Table 2).

### Robustness checks

**Fig. 2:**
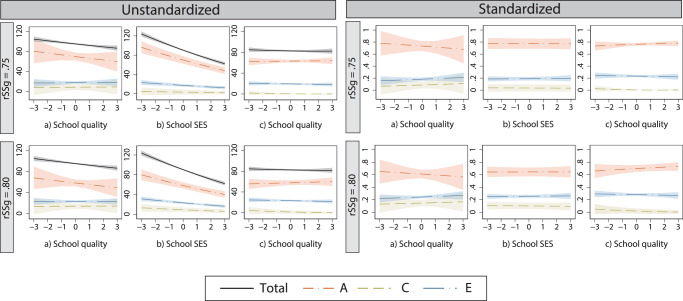
Unstandardized (left panel) and standardized (right panel) ACE moderations with alternative assumed genetic correlations of same-sex twins. Genetic (*A*), shared environmental (*C*), and non-shared environmental (*E*) variances of educational performance moderated by (**a**) school quality, (**b**) school SES, and (**c**) school quality controlled for the moderation effect of school SES and parental SES. Including 95% CI. Based on a model using a genetic correlation of *rSS*_*G*_ = 0.75 (top row) and *rSS*_*G*_ = 0.80 (bottom row).

First, we performed auxiliary analyses to test whether our findings were robust against using different values of genetic relatedness of same-sex twins. Conclusions based on our estimated genetic relatedness of *rSS*_*G*_ = 0.70 still hold when the alternative values 0.75 and 0.80 are used (Fig. 2, see also Supplementary information Appendix A). We still find no moderation of genetic and shared environmental variance by school quality. In our main analyses, we found a significant moderation of genetic variance by school quality when we did not control for school SES and parental SES. In our robustness check, this moderation effect by school quality is not statistically significant. As can be seen in Fig. 2 (and more detailed in Supplementary information Appendix A), the moderation effects, as well as the variance components in general, are estimated with less precision. Concerning the moderation by school SES, we still find a decreasing genetic variance with increasing school SES. When controlling for parental SES this negative moderation remains statistically significant and substantial when *rSS*_*G*_ = 0.75 is used (as was the case for our main results using *rSS*_*G*_ = 0.70), but not if *rSS*_*G*_ = 0.80 is used. Lastly, concerning our expectation that SES confounds the gene-school quality moderation effect, we found empirical support for this when using *rSS*_*G*_ = 0.70. For *rSS*_*G*_ = 0.75 and 0.80, we do not find statistically significant moderation effects of school quality in the first place, meaning there is no moderation effect that can be confounded by school SES and parental SES (Supplementary Tables 2, 3).

Second, we investigated whether our gene-environment interaction is driven by SES differences in the estimated MZ/DZ ratio among SS twins. We relied on the average estimated genetic relatedness of SS twins of *rSS*_*G*_ = 0.70 (and, alternatively, values of 0.75 and 0.80). However, given the factors that affect DZ twin pregnancies (e.g., IVF usage, maternal age, BMI, smoking), *rSS*_*G*_ may differ between SES groups. If there are relatively more DZ twins in higher-SES families than in lower-SES families, *rSS*_*G*_ will be lower in high-SES families than the assumed average of 0.70. Our observed gene-SES interaction could then be the result of an underestimation of genetic variance among twins from high-SES families (and an overestimation of genetic variance among lower-SES families). Our gene-SES interaction does not appear to be driven by SES differences in the MZ/DZ ratio. If anything, the estimated proportion of DZ twins among SS twins is larger for low-SES than for high-SES families. Our gene-SES interaction may therefore even be slightly underestimated.

Third, we investigated non-parametric gene-environment interactions for school quality and school SES. The *ACE-*moderation model including a continuous moderator assumes linear moderating effects on the *ACE* components, while there may be threshold effects (Purcell 2002). For example, it could be that only the most disadvantaged schools show increased genetic differences. Therefore, we performed a multigroup (i.e., non-parametric) gene-environment interaction for quantiles of school quality and school SES (see Supplementary Figs. 1–4). The results largely mirror the main analyses with continuous linear moderations. The only difference is that for school quality (not controlled for school SES and parental SES) the genetic variance is not declining linearly with increasing school quality. There is more genetic variance in the lowest-quality schools and less in the other four quantiles. Only the difference in genetic variance between the first and fourth quantile is statistically significant (Wald test = 5.78, *df* = 1, *p* = .016). For school SES, there is a clearer linear decline in genetic variance although not all group differences are statistically significant (see also the large confidence intervals in Supplementary Fig. 3).

Lastly, we used different operationalizations for our school quality variable (see Supplementary information Appendix C). The influence of certain more specific school quality aspects might be masked by using one overall school quality measure. We looked more specifically into the moderating role of school quality by separating it into the school resources and school climate dimensions (see Supplementary Table 10 for measurement details). We reach the same conclusion if we use these dimensions instead of one overall school quality factor. School resources and school culture are both positively associated with average educational performance, with a similar strength as the overall school quality factor (Supplementary Fig. 5). Similar to the main results, we find that school resources and culture negatively moderate genetic variance, but not when school SES and parental SES are controlled for (Supplementary Table 4). We also investigated all nine underlying school quality dimensions separately. When school SES is controlled for, only three dimensions remain statistically significantly associated with average performance (see Supplementary Fig. 6). These are guidance of educational needs, monitoring and evaluating (special needs) students, and learning climate. For these three dimensions, we performed moderation analyses. None of the dimensions moderates genetic variance. However, once we control for both school SES and parental SES, the remaining shared environmental variance turned out to be moderated by learning climate (see Supplementary Table 5 Model 2). We do not interpret this effect, because it is the only statistically significant school quality moderation we found out of many tests, and we did not correct *p*-values for multiple testing. Moreover, the effect is not substantial.

## Discussion

Inequality of educational opportunity is seen as a problematic phenomenon in many societies, making researchers, policymakers, and educational practitioners question how to reduce it. High-quality schools may especially have the potential to reduce educational inequality. We investigated this using gene-environment interaction analyses applied to administrative data on twins. Smaller shared environmental variance in higher-quality schools is indicative of less inequality of opportunity in these schools. Family background would then be less decisive for educational performance. Whether smaller genetic variance is also indicative of less inequality, is less straightforward. Genetic variance captures ‘positive’ and ‘negative’ potentials and there are different perspectives to what extent greater expression of such potentials in higher quality schools is considered to be fair or not.

We do not find evidence that school quality decreases educational inequality, neither concerning social inequality nor genetic inequality. Initially, there seemed to be some indication that genetic variance is smaller in higher-quality schools. However, the lower genetic variance in these schools appears not to be related to school quality but to school SES and parental SES instead. If genetic differences in performance are seen as unfair and part of educational inequality, there is less inequality in high-SES schools. We would have misattributed the decreasing genetic variance in educational performance to higher-quality school environments instead of higher-SES family and school environments (and overestimated the influence of school SES) if school quality, school SES, and parental SES were not studied simultaneously.

These findings thus suggest that not school quality but instead SES plays a role. The results suggest that it is both parental SES and school SES that matter and that the underlying mechanisms thus reflect processes in both the family and school context. The smaller genetic influence in higher-SES environments is consistent with the diathesis-stress model^9^. This model suggests that the fewer environmental risks and the more positive factors in higher-SES families and schools neutralize or compensate for the expression of genetic risks toward poor educational performance (see also Shanahan and Hofer 2005). Low-SES environments are generally considered less favorable for educational performance and may thus enforce such influences of genetic risk. This implies that if children have genetic risks (e.g., related to learning or behavioral problems), this will have fewer negative consequences for their educational performance if they have high-SES parents and attend high-SES schools. For example, high-SES parents may be more likely to provide adequate support. This is consistent with the sociological compensatory advantage mechanism, according to which prior negative outcomes (e.g., health and cognitive endowments at birth, previous school results) are compensated by high-SES parents^59^. Since such negative prior outcomes are genetically influenced, the compensatory advantage mechanism could be expanded to include the compensation of disadvantageous genetic dispositions. Similarly, a larger share of high-SES children in school may contribute to a more advantageous environment. For example, high-SES pupils may influence the performance, aspirations, and study habits of their peers and contribute to an environment that is more conducive to learning^56,60^. This may be especially beneficial for pupils with more genetic risks for lower performance.

The finding that both family SES and school SES moderate genetic variance suggests that low-SES children have a double disadvantage. Their genetic risks are less likely to be compensated in the family environment, and these children are also more often exposed to a (low-SES) school environment where compensation is less likely. Experiences in different contexts, including the family and school, are thought to combine and possibly interact with each other in shaping children’s educational outcomes (see ref. 7). Hence, it could be that an advantageous school environment may be especially beneficial for low-SES children. To gain more insight into the interrelated influences of families and schools, future research may incorporate interactions between the family and school to gain more insight into their combined effects on educational performance.

The decreasing genetic variance is accompanied by a decrease in the total variance in performance with increasing SES. It could be that pupils differ less in their performance levels in higher SES environments because genetic influences are compensated via the potential mechanisms that we just discussed. However, there could also be less variance in performance in high-SES environments because these environments are more homogenous in terms of children’s genetic makeup and/or environmental characteristics. In that case, focusing on the standardized results would be more appropriate. The standardized results do not show a gene-environment interaction. Hence, an alternative explanation for the lower genetic variance in high-SES schools is that selection into schools plays a role instead of a substantive interplay between genes and the school environment.

Although we had no expectations of moderation effects on non-shared environmental variance in performance, we find that it decreases with increasing SES. One interpretation of this finding may be that similar to the compensation of genetic risks, also non-shared environmental risks may be compensated in high-SES environments. Non-shared environmental risks include child-specific influences that negatively affect educational performance (e.g., accidents, illness, negative peer influences). If one twin has the risk of lower educational performance due to such individual circumstances, high-SES parents may be more likely to compensate for this^59,61^. Contrarily, low-SES parents may not have the opportunity to compensate (e.g., due to their lower levels of economic and cultural resources), hence, twins may end up performing differently. This would then be reflected in the larger non-shared environmental differences with lower SES. Since the non-shared environment also includes measurement error, an alternative interpretation is that there is less measurement error in the educational performance of high-SES twins. More research is needed for conclusions on the potential differential impact of the non-shared environment.

We did not find evidence for genetic and environmental influences being dependent on school quality, and only small effects of school quality on average educational performance. This could mean that the school environment is not as important for (inequality in) educational performance as thought. It may also be that there is less (systematic) variation in school quality in the Netherlands than in other countries because of how the educational context is organized. Private schools are rare and both public schools and religious schools receive public financing, proportional to the number of pupils. Schools attended by pupils from more disadvantaged backgrounds receive additional funding^62^. This could result in fewer quality differences between schools than in other countries where school funding is more unequal. In other contexts, the effects of school quality on average performance may be stronger and the gene-school quality interaction might work differently. More comparative research could provide insight into this, which may also provide a better understanding of the mixed findings of prior studies investigating gene-school environment interactions. It could also be that school quality matters, but that we do not sufficiently capture it with our indicators. For example, maybe it is not so much between-school quality differences but within-school differences that play a role (e.g., teacher quality, classroom processes). When differences in educational performance between twins that are in the same versus different classrooms are investigated, the classroom environment indeed turned out to play a role in the Netherlands^63^.

A potential limitation relates to the use of SS and OS twins. Although we use high-quality administrative data, a limitation is the absence of information on zygosity. This could also lead to biased estimates for genetic and shared environmental variance. Since the true genetic relatedness among SS twins is unknown, we had to rely on estimated genetic relatedness. To check how sensitive our results are to the model assumptions, we used different values for genetic relatedness among SS twins. We think our approach led to valid conclusions. Previous studies on educational performance for similar cohorts in the Netherlands, but based on a non-random twin sample with zygosity, found estimates for genetic, shared environmental, and non-shared environmental variance within the range of our estimates (i.e., 61–81% genetic, 0–15% shared environmental, and 9–24% non-shared environmental variance)^63–65^. Moreover, our conclusions relating to the gene-environment interactions remain the same irrespective of which value of genetic relatedness among SS twins is chosen.

The observed decrease in genetic differences and non-shared environmental differences in more advantageous environments is not entirely surprising, as a prior twin study on educational performance in the Netherlands found less unstandardized genetic variance (and less environmental variance) in educational performance with increasing family SES^44^. We show that this also holds for school SES. Also recent studies using polygenic PGIs provide evidence in line with this compensation pattern^27–29^. These findings can be used as a starting point for future research to investigate the mechanisms underlying the negative gene-SES interaction. The (decreasing) genetic differences in higher-SES environments as provided by the twin model, but also the educational attainment PGI, can be seen as a black box. They do not provide enough information on whether the genetic influence on educational performance occurs via characteristics that have a positive effect (e.g., cognitive ability) or negative effect (e.g., deviant behavior, psychiatric disorders). Future studies could identify mediators of the gene-SES interaction by investigating whether the gene-SES interaction in educational performance can be explained by gene-SES interactions in (non-)cognitive characteristics^66^. Empirically distinguishing between positive genetic potential and negative genetic risk would provide a more informative way to investigate whether the environment enhances genetic potential (i.e., bio-ecological model) or compensates genetic vulnerability (i.e., diathesis-stress model, compensatory advantage). If more advantageous environments compensate for genetic risks, it can be expected that this would be especially pronounced for (the genetic component of) more specific learning problems such as dyslexia and ADHD than (the genetic component of) general educational performance or cognitive ability, for example. This could be investigated by including such specific characteristics that have a negative effect on educational performance in the twin design and/or using the PGIs of these characteristics.

Both the twin design and usage of PGIs have advantages and disadvantages^67^. Therefore, providing a definite conclusion on the interplay between the school environment and genetic and shared environmental influences requires combining different methods. For now, based on our twin analyses, we conclude that school quality does not decrease (and neither increase) educational inequality. We find evidence for a gene-environment interplay in educational performance in the Netherlands, where genetic differences are smaller in more advantageous environments consistent with the idea of compensation for genetic risks. This gene-environment interplay in education turns out to be an SES composition effect rather than a school quality effect. Therefore, the results of this study suggest that reducing quality differences between schools would likely not be sufficient to reduce educational inequality.

If not only family background differences but also genetic differences are seen as a source of inequality that should be compensated, other policies may be more effective. Given the gene-school SES interaction observed in this study, it may be more worthwhile to reduce school segregation. These policies include, for example, changing school admission criteria and influencing the school choice behavior of parents. Additionally, the way schools are funded could be reformed. Currently, primary schools receive additional funding if they have a larger share of pupils from disadvantaged family backgrounds^62^. This could be extended to additional funding to compensate for genetic risks. Since there are practical and ethical concerns with directly measuring genetic risks, measured expressions of these risks (e.g., low cognitive ability, dyslexia, ADHD) could be used as proxies. Such policies may contribute to more equal chances to learn and perform well in school for all children, including those with genetic risks towards lower performance.

## Methods

### Data

We use linked microdata from Statistics Netherlands (*CBS*) covering the whole population. We construct a dataset including twin families with information on children’s educational performance, school environment, and family SES. To construct twin families, we rely on basic demographic information on children and their legal parents, using linked parent-child data (*KINDOUDERTAB*, see ref. 68) combined with the municipal personal records database (*GBAPERSOONTAB*, see ref. 69). We identify families based on children who share the same legal parents. After constructing families, we identify twin pairs. Since the birth day is not available because of privacy reasons, we base this on children who have the same birth month and year. Based on the sex composition, we identify same-sex (SS) and opposite-sex (OS) twin pairs. Multiple twin pairs in one family are analytically complex. Therefore, we select one random twin pair in these cases.

We use the *Cito* database (*CITOTAB*, see ref. 70) to obtain information on educational performance. These data are available for 2006–2019 (birth cohorts 1994–2007) at the time of this study. Primary schools can permit *Cito* to share the data with the *CBS*, who anonymized the data and assigned identification numbers to link the data at the individual and school level. Data sources on parental SES that we use to construct school SES are the highest education database (*Hoogsteopltab*, see ref. 71) for the year 2018 and personal income for the period 2003–2018 (*IPI* for 2003–2015 and *INPATAB* for 2011–2018, see ref. 72). Data on educational attainment are largely based on diverse registrations of individuals who completed their education at an educational institution funded by the government. There is no (reliable) register data available for privately funded education (which is relatively rare in the Netherlands), education abroad, and long-term corporate training. To add information on this, the *CBS* used data from the Labor Force Survey which is collected on a sampling basis. Income data is based on administrative information, mostly provided by the tax authorities.

We supplement children and parent data from *CBS* with official information on the school environment obtained from the Dutch Inspectorate of Education and the Dutch Education Executive Agency. Inspectorate of Education data include many indicators that are used to assess the quality of schools by the inspectorate and are generally available from 1999 onward. Education Executive Agency data include information on general school characteristics such as the number of students and teachers. These data are (mostly) available from 2011 onward. School data can be linked to the *CBS* data via a school identifier (*BRIN*). This research was approved by the Ethics Committee of the Faculty of Social and Behavioral Sciences, Utrecht University (FETC20-216). Given the usage of administrative data, informed consent is not applicable.

### Selections and selectivity

**Fig. 3:**
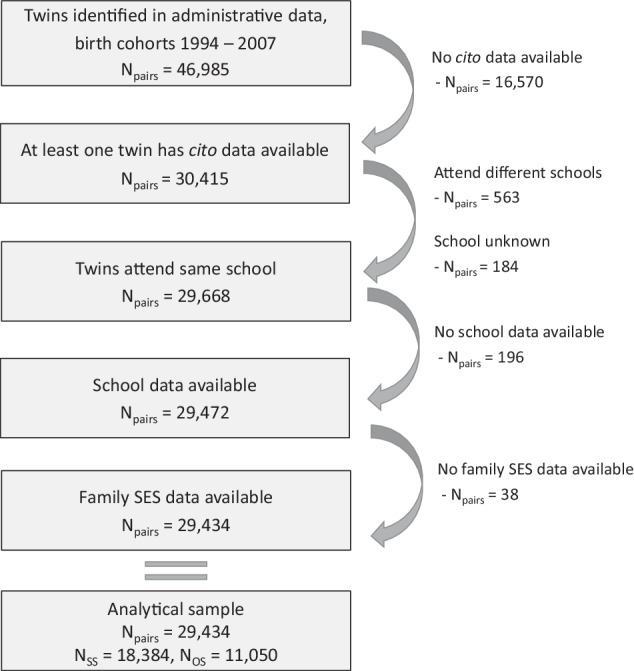
Selection of the analytical sample. SS same-sex twin pairs, OS opposite-sex twin pairs.

Figure 3 shows the sample selection. We only study twins from birth cohorts 1994–2007, due to data availability of our dependent variable. Only twin pairs for whom at least one twin has information on educational performance are included. One reason for the missingness is that some schools did not permit to share the results with *CBS*. Another reason is that schools can choose to administer another test. Most schools use the *Cito* test. Until recently, around 80% of the schools administered this test and these schools did not differ from the total school population regarding region, school size, urbanization, and percentage of students from low-educated families^73^. For the most recent years, the percentage of schools administering the *Cito* test decreased (63.8% in 2017/2018, 55.9% in 2018/2019) and became a bit more selective. Schools in more urbanized areas and larger schools more often administered the *Cito* test^73,74^. Excluding these years did not substantially change our results.

We only include twins who went to the same primary schools. Most twins (in our data 98%) go to the same school. Those who attend different schools form a selective group (e.g., one twin attends a school for special needs). For 6415 twin pairs, at least one of the twins had missing information on the school identifier. In most of these pairs (6234 pairs), there was a co-twin with non-missing information. In these cases, we assume that both twins attended the same school. We exclude the twin pairs where both twins had missing information on school data. We also exclude twin pairs with missing information on parental SES. This leads to our analytical sample of *N*_*pairs*_ = 29,434.

### Measurements

We measure our dependent variable, *educational performance*, by students’ scores on the *Cito* test. We use the most recent score in case of multiple observations, which may occur if children repeat the grade when the test is taken. The *Cito* test is a nationwide standardized educational achievement test taken at the end of primary education around age 12. It consists of multiple-choice items on Dutch language, mathematics, study skills, and world orientation (e.g., geography, biology, and history). The domains are combined into a standard score. Because the subdomain world orientation is not mandatory, this is not included in the standard score. The standard score is calculated based on the correctly answered questions with a formula taking into account the difficulty of the test for that year, to make the scores comparable over the years. It is aimed to have questions of a difficulty level between 0.40 and 0.90 with an average of around 0.70, where the difficulty level refers to the proportion of pupils who answered the item correctly^73^. Another property of the *Cito* score is that the number of correct answers is transformed into a scale from 501–550, with a national average of 535 and a standard deviation of 10. If the scores are below 501 or above 550, they are rounded to the minimum or maximum^73^. There is more censoring at the upper end than at the lower end of the scale. These properties of the scaling result in a somewhat negatively skewed distribution, although the skewness value of −0.6 does not suggest severe skewness. Also, the level of censoring is relatively low, with around 4% of twins obtaining the maximum score of 550. However, censoring occurs more among high-SES children (and thereby also more frequently in high-SES and high-quality schools). High-SES children more often obtain the maximum score, while obtaining the lowest score is uncommon. This pattern is also found in similar studies (see, e.g., refs. ^44,75^) and has the consequence that gene-environment interactions may be (partly) due to a ceiling effect. We expect this to be a limited problem in our study. Both the *Cito* score and the (uncensored) raw test score show decreasing variance with increasing parental SES (see Supplementary Fig. 7). Also, a prior study investigating gene-family SES interactions in *Cito* scores shows that correcting for censoring did not change the results^44^. Hence, it is unlikely that the decreasing variance with increasing parental SES (and the associated measures school SES and school quality) is solely driven by the censoring of the *Cito* scale.

For our moderator *school quality*, we construct a factor score based on data from the Inspectorate of Education. The Inspectorate data consist of many official indicators that are used to assess the quality of schools. These are generally available for the period 2000–2011, and sometimes up to 2019. Initially, we also included data from a second data source, namely, data from the Education Executive Agency. However, in the end, the indicators derived from these data (e.g., financing, number of pupils and staff) were not included in our measurement model due to low correlations with the other indicators or low factor scores (see Supplementary information Appendix E). The inspectorate data are not collected for scientific research purposes, but to assess whether schools meet a certain quality standard. The inspectorate usually visited schools once every four years, and the set of indicators that were used differed over the years. Although this provides a rich source of information, these data are not directly suited for research and require extensive data handling. The structure of the Inspectorate data with the resulting missing data makes it impossible to measure school quality per year or even a couple of years. Therefore, for each indicator, we take the average of all available years. Items are mostly measured on a three-point scale (insufficient, sufficient, good) or a four-point scale (bad, insufficient, sufficient, good). Sometimes, also a two-point scale is used (insufficient, sufficient; no, yes).

We construct factor scores based on the standardized items using factor analyses with Full Information Maximum Likelihood (FIML) estimation in Mplus. We conduct two Exploratory Factor Analyses (EFA): one for all the items related to school resources leading to two dimensions, and one for school climate leading to seven dimensions. Altogether, this leads to nine dimensions of school quality: (1) range of educational activities, (2) (implementation of) school curriculum, (3) guidance of educational needs, (4) parental involvement, (5) monitoring and evaluating (special needs) students, (6) learning climate, (7) social climate, (8) safety, (9) quality assurance (see Supplementary Tables 6, 7). Based on these dimensions, we construct one overall school quality factor in a third (confirmatory) factor analysis. The dimensions of social climate, parental involvement, and safety have a low loading on this overall factor (Supplementary Table 8) and/or a low correlation with schools’ average *Cito* score (Supplementary Table 9). As an alternative operationalization, we exclude these dimensions and construct a factor score based on the remaining six dimensions. This does not lead to substantially different results and therefore we keep all the dimensions in. Given the numerous latent variables and items, it is not possible to integrate the full measurement model with our analytical model. Therefore, we save the factor scores and include these in our analytical model as a single variable while imposing a measurement error correction. More information on this correction – as well as further details on the procedure, items, and factor analyses – are provided in the Supplementary information (Appendix E).

*Parental SES* is measured by a factor score based on parental education and income. For parental education, we use the father’s and mother’s highest attained level of education, which is coded according to the International Standard Classification of Education (ISCED) 2011. We rely on the most recent data file from 2018. For income, we use the father’s and mother’s percentile scores of personal yearly income for the year before the *Cito* test. Personal income includes the gross income from labor, own company, income insurance benefits, and social security benefits (excluding child benefits and child-related budget). Premiums for income insurance have been deducted. For the percentile score, personal income is divided into 100 equal groups of people with income in private households. We construct a factor score for SES based on standardized items using CFA in Mplus (see Supplementary information Appendix F). FIML is used to handle missing data, which is especially present for parental education (Supplementary Table 11). Data on parental income is also sometimes missing (for the father’s and mother’s income in 5% and 12% of the cases, respectively). We have income data on fathers and mothers who no longer form a household. Hence, missing values are not caused by a divorce or separation but the result of a combination of unknown causes. These potentially include a deceased parent, a migrated parent, a parent without income, or a data registration problem. Given the unknown causes and relatively small amount of missing, using FIML estimation in the parental SES measurement model is a suitable solution.

*School SES* is an aggregate of parental SES. We use the average parental SES of all children in the school who took the *Cito* test in the year that the twins took this test.

We control for *year of birth* and *sex* (0 = female, 1 = male) in all models. Descriptive statistics of all variables are presented in Table 3.

**Table 3:** Descriptive statistics for same-sex (SS) and opposite-sex (OS) twins

	SS twins			OS twins		
Variable	*N*	Mean	S.D.	*N*	Mean	S.D.
*Twin specific*						
Cito twin-1	16,530	535.17	9.77	9909	535.08	9.82
Cito twin-2	16,456	535.32	9.68	9909	535.13	9.73
Male twin-1	18,384	0.49		11,050	0.50	
Male twin-2	18,384	0.49		11,050	0.50	
*Twin pair*						
School quality	18,384	0.10	0.46	11,050	0.10	0.45
School SES	18,384	0.03	0.34	11,050	0.03	0.34
Parental SES	18,384	0.06	0.77	11,050	0.04	0.78
Year of birth	18,384	2000.12	3.73	11,050	2000.19	3.68

All continuous independent variables are *z*-standardized prior to the analyses. Minimum and maximum values are not provided because of the confidentiality guidelines of Statistics Netherlands.

### Twin method

**Fig. 4:**
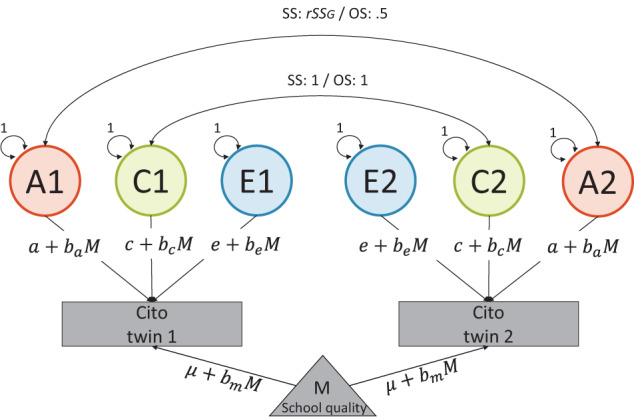
ACE moderation model. Latent variables represent genetic (*A*), shared-environmental (*C*), and non-shared environmental (*E*) components of educational achievement, with corresponding path coefficients (a, c, e). Measured variable *M* refers to the moderator. Genetic covariance for same-sex (SS) twin pairs is estimated by ${{rSS}}_{G}=1\frac{{N}_{{SS}}-{N}_{{OS}}}{{N}_{{SS}}}+0.5\frac{{N}_{{OS}}}{{N}_{{SS}}}$ = 0.70 and is 0.50 for opposite-sex (OS) twin pairs. We also use 0.75 and 0.80 as alternative values for *rSS*_*G*_.

In the classical twin design, structural equation modeling (SEM) is used to decompose the variance in a characteristic into three latent components. First, there is a component capturing additive genetic variance (*A*). This component partly reflects genetically influenced traits that positively influence educational performance, including cognitive ability but also non-cognitive traits such as self-efficacy and grit^76^. It also captures genetic variance related to traits that negatively influence performance, for example, ADHD, depression, and antisocial behavior^20,77^. Second, there is a common or shared environmental variance (*C*) component, which includes all environmental aspects making twins more alike such as influences of family resources, parenting practices, educational expectations, and the broader environmental context that differs between families^40^. We use the *C-*component as a comprehensive measure of family background influences, reflecting social inequality in educational performance. Last, there is unique, non-shared, environmental variance (*E*), capturing aspects that make twins dissimilar. These include, for example, subjective experiences, differential treatments, luck, and measurement error^21^. Latent components *A*, *C*, and *E* are set to a variance of 1. Path coefficients a, c, and e represent the effects of the latent factors on educational performance. The variance is equal to the square of the path coefficient; hence the *ACE* model can be written mathematically as

1 $${V}_{{educ}}={a}^{2}+{c}^{2}+{e}^{2}={V}_{A}+{V}_{C}+{V}_{E}$$

where ${V}_{{educ}}$ is the total variance of our phenotype educational performance. We extend this model by including a continuous moderator (*M*)^78^. In our case, our moderator school quality affects the average educational performance as shown by $\mu +{b}_{m}M$. It could also moderate, for example, *a* to become $a+{b}_{a}M$ (see Fig. 4). The total variance in this moderation model changes to

2 $${V}_{{educ}{\rm{|}}M}={\left(a+{b}_{a}M\right)}^{2}+{\left(c+{b}_{c}M\right)}^{2}+{\left(e+{b}_{e}M\right)}^{2}$$

Parameters in this model are usually identified because zygosity is available. Identical (i.e., monozygotic; MZ) and fraternal (i.e., dizygotic; DZ) twins differ in genetic resemblance: where MZ twins are genetically identical at conception, DZ twins share on average half of their segregating genes. Hence, the genetic correlation (*A1*-*A2*) can be constrained to 1 for MZ twins and 0.5 for DZ twins. It is assumed that MZ and DZ twins share their environment to the same extent, meaning that shared environmental correlation (*C1*-*C2*) can be constrained to 1 for both MZ and DZ twins. Accordingly, the MZ covariance is ${{Cov}}_{{mz}}={V}_{A}+{V}_{C}$ and for DZ twins this is ${{Cov}}_{{Dz}}={0.5V}_{A}+{V}_{C}.$

Constraining the shared environmental variance to be equal reflects the Equal Environment Assumption (EEA). Violation of the EEA could overestimate *A* and underestimate *C*, but only if differential treatment is related to the outcome under study. Several studies showed that the EEA is unproblematic for a wide range of outcomes^79^, including school performance specifically^80,81^. Additional assumptions are no assortative mating, generalizability of twins to the general population, minimal gene-environment correlation, and absence of non-additive genetic effects. Violations of these assumptions could bias *A* and *C* but do so in different directions^82^. These consequences of violations (upward or downward bias of *A* and *C*) are reflected in the difference in genetic relatedness between twin types. We perform the analyses with different genetic correlations to test to what extent our results are sensitive to the assumptions.

### Twin model with unknown zygosity

We do not have information on zygosity but instead rely on data from 18,384 same-sex (SS) and 11,050 opposite-sex (OS) twin pairs. OS twins are always DZ, hence their genetic correlation (*A1*-*A2*) is equal to 0.5. SS twins are a mixture of MZ and DZ twins. The true value for the average genetic correlation of SS twins (i.e., *rSS*_*G*_) is unknown, and there are different ways in the literature to deal with this^32,83,84^. A common way is using Weinberg’s differential rule to estimate the proportion of MZ and DZ twins within SS twin pairs^85^. According to this rule, the probability of male births equals the probability of female births, and therefore among DZ twins the number of SS twins equals the number of OS twins. The total number of DZ twins is thus twice the number of OS twins. The proportion of MZ twins within SS pairs in our data can be estimated by ${p}_{{MZSS}}$ = (*N*_*SS*_ – *N*_*OS*_) / *N*_*SS*_ = (18,384–11,050) / 18,384 = 0.40. For DZ twins within SS pairs this is ${p}_{{DZSS}}$ = 1 – 0.40 = 0.60. This leads to an average genetic relatedness among SS twins of *rSS*_*G*_ = (1*0.40) + (0.5*0.60) = 0.70. Another common approach, which is based on the assumption that among SS twins half will be MZ and half will be DZ, is to use ${{rSS}}_{G}$ = 0.75 (i.e., the average genetic relatedness of MZ and DZ twins)^84^.

While the MZ twin rate is relatively stable over time and MZ twinning is thought to be the result of a random event, this is not the case for DZ twins. DZ twin births are related to individual characteristics (DZ twin pregnancies are more common when the mother is older, taller, has a higher BMI, and smokes, among others) and the usage of assisted reproductive technology (ART) such as in vitro fertilization (IVF)^86^. There are also indications that the usage of ART is related to MZ twin pregnancies, but the underlying causes are unknown^86,87^. In the Netherlands, the average maternal age and use of ART increased over the past decades, although the IVF policy has become more conservative (increasingly only one embryo is being transferred)^86^. Assuming a fifty-fifty mixture of MZ and DZ twins among the SS pairs is likely not realistic for the population that we study. Relying on the estimated genetic relatedness using Weinberg’s differential rule overcomes this problem. Indeed, when we calculate the MZ/DZ ratio among SS twins we find a larger share of DZ twins among SS twins (i.e., a ratio of 40/60, leading to the estimated genetic relatedness of *rSS*_*G*_ = 0.70).

Relying on a twin model with unknown zygosity has been criticized^88^. One concern is that the design is less powerful than using information on zygosity. This is less applicable to our study given the use of population data. Another concern is that the method relies on the assumption that the correlation of SS twin pairs only differs from that of OS twin pairs because SS twins are on average genetically more similar, not for other non-genetic reasons^32^. This assumption is violated if SS DZ twins are more similar to one another than OS DZ twins because the first are from the same sex and the latter are not. One could test this by comparing intraclass correlation coefficients (ICCs) of SS and OS pairs with known zygosity. This has been done for reading and mathematics achievement using data from the Netherlands Twin Register^45^. Comparing the similarity in educational achievement for SS and OS pairs and MZ and DZ pairs with different sex compositions demonstrated that the assumption holds^45^. Another way to test this is by comparing the ICCs of SS and OS non-twin sibling pairs. This shows that SS siblings are slightly more similar (average ICC for males and females = 0.44) than OS siblings (ICC = 0.42), suggesting small sex influences (Supplementary Fig. 8).

Although the difference is only 0.02, it could still lead to a non-negligible upward bias in estimates of genetic variance and a downward bias in shared environmental variance. To give an intuition for the possible size of the bias, if one uses the ICC of OS DZ twins (0.45), the descriptive estimate of heritability would be 0.80, which can be calculated with the formula (ICC_SS_ –ICC_OS_) / (*rSS*_*G*_ – *rOS*_*G*_) = (0.61–0.45) / (0.70–0.50). If we assume that the sex-effect for twins is the same as for non-twin siblings, the ICC for SS DZ twins would be 0.45 + 0.02 = 0.47. Based on this ICC, heritability would be 0.70. One can correct this bias by using a larger value for *rSS*_*G*_. Note that theoretically, one would adjust the shared environmental correlation of OS twins downwards to consider that their environments are less similar because they are of different sexes. However, genetic and shared environmental relatedness account for the same pattern in the data (cf. Spinath et al. 2004), so it makes sense to only adjust one at a time. Practically, increasing *rSS*_*G*_ is similar to decreasing the shared environmental correlation of OS twins. We, therefore, perform our analyses using three values of *rSS*_*G*_ (0.70, 0.75, 0.80). As we will show, our conclusions are robust to the different values.

### Analytical strategy

We fit a series of *ACE* models in Mplus. We have data on 29,434 twin pairs nested in 5843 schools. To account for this nested structure, we adjust the standard errors for clustering at the school level. In all models, the influences of sex and birth year are controlled for by including them as covariates. We *z*-standardize all continuous independent variables prior to the analyses. Before fitting the twin models, we test for equal means and variances between SS and OS twins. The difference in the mean of educational performance (Wald test = 0.83, *df* = 1, *p* = 0.362) and variance in educational performance (Wald test = 0.12, *df* = 1, *p* = 0.733) are not statistically significant, indicating that equality of means and variances can be assumed.

We first examine the *ACE* model and include the main effects of school quality, school SES, and parental SES on educational performance in a stepwise fashion. It should be noted that these school and family measures are always shared between twins and thus can only explain shared environmental variance in the *ACE* model, even though these measures include genetic and non-shared environmental variability^89^. Hence, their associations with educational performance should not be interpreted as causal, as they can be genetically confounded^90^. Next, we allow the *ACE* components to be moderated by school quality and school SES to test whether genetic and shared environmental variance in educational performance increases or decreases with increasing school quality and school SES. Subsequently, we test the moderation by school quality and school SES simultaneously, to see whether school SES explains part of the moderation effect of school quality. Lastly, we control for the moderation by parental SES to further scrutinize whether moderation by the school environment measures reflects school-based processes or are instead driven by what happens in the family environment. We perform several robustness checks to assess to what extent our results are dependent on our model assumptions and operationalization of school quality.

The few behavioral genetics studies examining whether the school environment moderates genetic and shared environmental variance generally looked at absolute variance components^23–25^, although standardized components were also used^22^. For standardized components, each variance component is made proportional to the total variance. For example, relative genetic contribution (i.e., heritability) is obtained by $S{V}_{A}=\frac{{V}_{A}}{{V}_{{educ}}}$. An advantage of standardized components is that it considers that the total variance may differ between contexts while the effect of genes and the environment do not differ. For example, in certain schools children may be more genetically similar or more similar concerning their (non-)shared environmental background than in other schools^64^. An advantage of using unstandardized, absolute variance components is that genetic and shared environmental variances can be contingent on school quality independent of each other. Solely focusing on standardized components will conceal underlying processes. As both standardized and unstandardized variance components have pros and cons, we report both.

### Reporting summary

Further information on research design is available in the Nature Research Reporting Summary linked to this article.

### Supplementary information

Supplemental Material

Reporting summary

## Data Availability

All results are based on own calculations using non-public microdata from Statistics Netherlands. Under certain conditions, these microdata are accessible for statistical and scientific research. For further information, see https://www.cbs.nl/en-gb/onze-diensten/customised-services-microdata/microdata-conducting-your-own-research or e-mail microdata@cbs.nl. Summary data of the variables used in this study can be found here: https://osf.io/xsgdt/.

## Code availability

Code for replication can be found here: https://osf.io/xsgdt/.
