# Supplementary material for: Gene-environment interaction analysis of school quality and educational inequality
Source: NPJ Sci Learn. 2024 Mar 1;9:14. doi: 10.1038/s41539-024-00225-x (PMC10907386; doi:10.1038/s41539-024-00225-x)
Supplement: Supplementary file 1 — Supplemental Material [file 41539_2024_225_MOESM1_ESM.pdf]

Supplementary Material

Gene-environment interaction analysis of school quality and educational inequality

Kim Stienstra[1,*] Antonie Knigge[1] & Ineke Maas[1, 2]

[1] Department of Sociology/ICS, Utrecht University, The Netherlands

[2] Department of Sociology, Vrije Universiteit Amsterdam, The Netherlands

* Corresponding author

Email: Kim.Stienstra@eui.eu

ORCID: 0000-0002-9877-621

Index

Appendix A. Additional results for different values of rSS_G 3

Supplementary Table 1. 3

Supplementary Table 2. 5

Supplementary Table 3 6

Appendix B. Non-parametric gene-environment interactions 7

Supplementary Figure 1. 7

Supplementary Figure 2. 7

Supplementary Figure 3 8

Supplementary Figure 4. 8

Appendix C. Results school quality dimensions 9

Supplementary Figure 5. 9

Supplementary Table 4 10

Supplementary Figure 6. 11

Supplementary Table 5 12

Appendix D. Censoring 13

Supplementary Figure 7. 13

Appendix E. Measurement model school quality 14

Supplementary Table 6. 16

Supplementary Table 7 17

Supplementary Table 8 19

Supplementary Table 9 20

Supplementary Table 10 21

Appendix F. Measurement model socioeconomic status (SES) 22

Supplementary Table 11 22

Appendix G. Intraclass Correlation Coefficients 23

Supplementary Figure 8. 23

Appendix A. Additional results for different values of rSS_G

Supplementary Table 1. *ACE model for cito with main effects of school quality and school SES for different values of* rSS_G *(*$N_{SSpairs}$ *= 18,384,* $N_{OSpairs}$ *=11,050).*

	rSS_G = .70						rSS_G = .75					
	Model 1		Model 2		Model 3		Model 1		Model 2		Model 3	
**Parameter**	**Estimate**	**s.e.**	**Estimate**	**s.e.**	**Estimate**	**s.e.**	**Estimate**	**s.e.**	**Estimate**	**s.e.**	**Estimate**	**s.e.**
Intercept	534.63***	(0.08)	534.64***	(0.07)	534.65***	(0.07)	534.63***	(0.08)	534.64***	(0.07)	534.65***	(0.06)
a	9.28***	(0.06)	8.91***	(0.06)	8.33***	(0.06)	8.34***	(0.24)	8.34***	(0.23)	8.08***	(0.06)
c	0.00	(0.00)	0.00	(0.00)	0.00	(0.00)	2.85***	(0.48)	1.89**	(0.71)	0.00	(0.00)
e	2.98***	(0.12)	3.35***	(0.10)	3.82***	(0.09)	4.16***	(0.16)	4.16***	(0.16)	4.31***	(0.07)
V_A	86.16***	(1.14)	79.29***	(1.08)	69.40***	(1.03)	69.61***	(3.95)	69.50***	(3.91)	65.32***	(0.96)
V_C	0.00	(0.00)	0.00	(0.00)	0.00	(0.00)	8.13**	(2.73)	3.57	(2.67)	0.00	(0.00)
V_E	8.86***	(0.71)	11.20***	(0.70)	14.59***	(0.69)	17.27***	(1.32)	17.32***	(1.31)	18.56***	(0.64)
School quality	0.61***	(0.07)	0.25***	(0.06)	0.24***	(0.06)	0.61***	(0.07)	0.25***	(0.06)	0.24***	(0.06)
School SES			2.19***	(0.06)	0.92***	(0.06)			2.19***	(0.06)	0.92***	(0.06)
Parental SES					2.89***	(0.05)					2.89***	(0.05)
Freely estimated parameters	10		11		12		10		11		12	
Loglikelihood	-232015.70		-231114.62		-229734.35		-232015.68		-231112.87		-229725.54	
Scaling correction factor	2.57		2.38		2.28		2.70		2.50		2.29	
AIC	464051.41		462251.23		459492.70		464051.36		462247.74		459475.08	

Note: * $p < .05$, ** $p < .01$, *** $p < .001$ (two-tailed test). Controlled for sex and year of birth. All continuous independent variables are z-standardized prior to the analyses. Robust standard errors accounting for clustering at the school level are shown in parentheses. Parameters *a*, *c*, and *e* refer to unmoderated path coefficients capturing genetic, shared-environmental, and non-shared environmental influences, respectively. The *b* coefficients refer to the moderation effects of *a, c*, and *e*, by school quality (i.e., resources or culture), school SES, and parental SES.

Supplementary Table 1. *(continued)*.

	rSS_G = .80					
	**Model 1**		**Model 2**		**Model 3**	
**Parameter**	**Estimate**	**s.e.**	**Estimate**	**s.e.**	**Estimate**	**s.e.**
Intercept	534.63***	(0.08)	534.64***	(0.07)	534.66***	(0.07)
a	7.62***	(0.22)	7.61***	(0.22)	7.61***	(0.21)
c	3.73***	(0.32)	3.06***	(0.39)	1.66*	(0.70)
e	4.80***	(0.11)	4.81***	(0.11)	4.81***	(0.10)
V_A	58.01***	(3.30)	57.93***	(3.27)	57.96***	(3.22)
V_C	13.92***	(2.42)	9.36***	(2.37)	2.76	(2.33)
V_E	23.07***	(1.02)	23.11***	(1.01)	23.11***	(1.00)
School quality	0.61***	(0.07)	0.25***	(0.06)	0.24***	(0.06)
School SES			2.19***	(0.06)	0.92***	(0.06)
Parental SES					2.89***	(0.05)
Freely estimated parameters		10		11		12
Loglikelihood		-232015.68		-231112.87		-229724.67
Scaling correction		2.70		2.50		2.40
AIC		464051.36		462247.74		459473.35

Note. * $p < .05$, ** $p < .01$, *** $p < .001$ (two-tailed test). Controlled for sex and year of birth. All continuous independent variables are z-standardized prior to the analyses. Robust standard errors accounting for clustering at the school level are shown in parentheses. Parameters *a*, *c*, and *e* refer to unmoderated path coefficients capturing genetic, shared-environmental, and non-shared environmental influences, respectively. The *b* coefficients refer to the moderation effects of *a, c*, and *e*, by school quality (i.e., resources or culture), school SES, and parental SES.

Supplementary Table 2. *ACE model for cito with interaction effects of school quality, school SES, and parental SES for* $\boldsymbol{rSS_G}$ ***= .75*** *($N_{SSpairs}$ = 18,384, $N_{OSpairs}$ =11,050).*

	**Model 1**		**Model 2**		**Model 3**		**Model 4**	
**Parameter**	**Estimate**	**s.e.**	**Estimate**	**s.e.**	**Estimate**	**s.e.**	**Estimate**	**s.e.**
Intercept	534.63***	(0.08)	534.65***	(0.07)	534.65***	(0.07)	534.67***	(0.06)
a	8.35***	(0.23)	8.36***	(0.24)	8.36***	(0.23)	7.95***	(0.08)
$b_a SQ$	-0.23	(0.20)			-0.18	(0.11)	0.01	(0.07)
$b_a SchoolSES$			-0.48***	(0.05)	-0.44***	(0.06)	-0.27**	(0.08)
$b_a ParentalSES$							-0.63***	(0.08)
c	2.83***	(0.47)	1.79**	(0.76)	1.70*	(0.80)	0.43	(0.37)
$b_c SQ$	0.07	(0.40)			0.48	(0.38)	-0.31	(0.26)
$b_c SchoolSES$			-0.13	(0.09)	-0.24	(0.14)	-0.93***	(0.21)
$b_c ParentalSES$							0.77***	(0.18)
e	4.15***	(0.16)	4.15***	(0.16)	4.15***	(0.15)	4.39	(0.08)
$b_e SQ$	0.04	(0.14)			0.08	(0.09)	-0.04	(0.07)
$b_e SchoolSES$			-0.22**	(0.07)	-0.24**	(0.07)	-0.09	(0.08)
$b_e ParentalSES$							-0.25**	(0.08)
School quality	0.61***	(0.07)			0.24***	(0.06)	0.22***	(0.06)
School SES			2.23	(0.06)	2.20***	(0.06)	0.91***	(0.06)
Parental SES							2.94***	(0.05)
Freely estimated parameters	13		10		17		21	
Loglikelihood	-217429.87		-174627.56		-216382.04		-214768.90	
Scaling correction factor	2.36		1.14		2.00		1.80	
AIC	434885.74		349275.12		432798.07		429579.81	

Note. **p*<.05, ***p*<.01, ****p*<.001 (two-tailed test). A genetic correlation of rSS_G=.75 is used. Controlled for sex and year of birth. All continuous independent variables are z-standardized prior to the analyses. Robust standard errors accounting for clustering at the school level are shown in parentheses. Parameters *a*, *c*, and *e* refer to unmoderated path coefficients capturing genetic, shared-environmental, and non-shared environmental influences, respectively. The *b* coefficients refer to the moderation effects of *a, c*, and *e*, by school quality (SQ), school SES, and parental SES.

Supplementary Table 3. *ACE model for cito with interaction effects of school quality, school SES, and parental SES for* ***rSS_G = .80*** *($N_{SSpairs}$ = 18,384, $N_{OSpairs}$ =11,050).*

	Model 1		Model 2		Model 3		Model 4	
**Parameter**	**Estimate**	**s.e.**	**Estimate**	**s.e.**	**Estimate**	**s.e.**	**Estimate**	**s.e.**
Intercept	534.63 ***	(0.08)	534.65 ***	(0.07)	534.65***	(0.07)	534.67***	(0.06)
a	7.61***	(0.22)	7.61 ***	(0.21)	7.63***	(0.22)	7.61***	(0.15)
b_aSQ	-0.19	(0.21)	-0.69 ***	(0.05)	-0.20	(0.19)	0.04	(0.09)
$b_aSchoolSES$					-0.39***	(0.08)	-0.13	(0.12)
$b_aParentalSES$							-0.72***	(0.10)
c	3.73***	(0.32)	1.80 **	(0.65)	2.98***	(0.41)	1.21	(0.65)
b_cSQ	-0.01	(0.31)	-0.12	(0.08)	0.30	(0.40)	-0.26	(0.27)
$b_cSchoolSES$					-0.27	(0.14)	-1.03***	(0.18)
$b_cParentalSES$							0.76***	(0.19)
e	4.80***	(0.11)	4.81 ***	(0.10)	4.80***	(0.11)	4.82***	(0.08)
b_eSQ	-0.01	(0.10)	-0.34 ***	(0.06)	0.06	(0.10)	-0.05	(0.07)
$b_eSchoolSES$					-0.28***	(0.06)	-0.16*	(0.08)
$b_eParentalSES$							-0.24***	(0.07)
School quality	0.61***	(0.07)			0.24***	(0.06)	0.22***	(0.06)
School SES			2.23 ***	(0.06)	2.20***	(0.06)	0.91***	(0.06)
Parental SES							2.94***	(0.05)
Freely estimated parameters	13		10		17		21	
Loglikelihood	-217429.86		-173219.29		-216382.12		-214766.43	
Scaling correction factor	2.37		1.12		2.03		1.85	

Note. *p<.05, **p<.01, ***p<.001. A genetic correlation of rSS_G=.80 is used. Controlled for sex and year of birth. All continuous independent variables are z-standardized prior to the analyses. Robust standard errors accounting for clustering at the school level are shown in parentheses. Parameters *a*, *c*, and *e* refer to unmoderated path coefficients capturing genetic, shared-environmental, and non-shared environmental influences, respectively. The *b* coefficients refer to the moderation effects of *a, c*, and *e*, by school quality (SQ), school SES, and parental SES.

Appendix B. Non-parametric gene-environment interactions

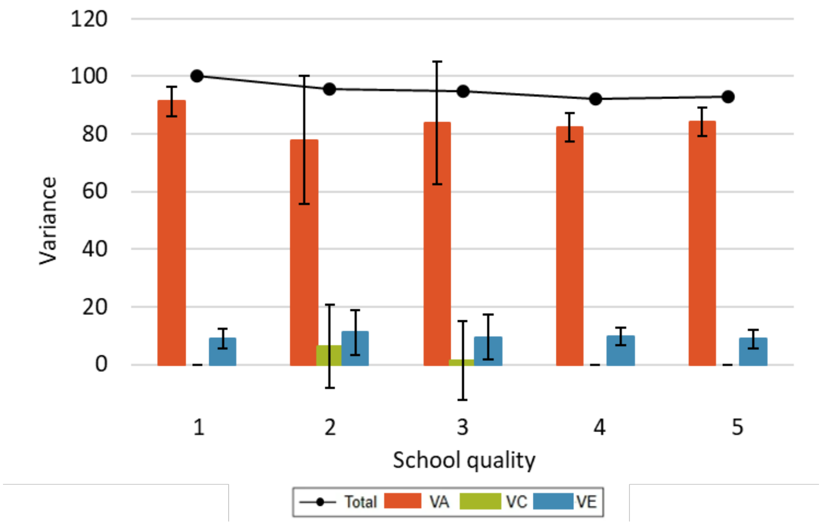

Supplementary Figure 1. Unstandardized genetic (*A*), shared environmental (*C*), and non-shared environmental (*E*) variances of educational performance moderated by quintiles of school quality in a non-parametric gene-environment interaction analysis, including 95% CI.

Note. Based on a model using a genetic correlation of rSS_G=.70, controlled for sex and year of birth.

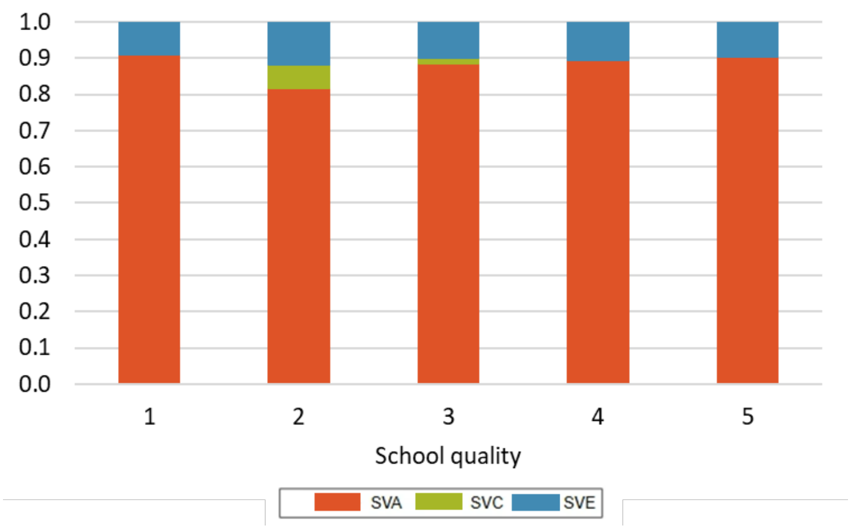

Supplementary Figure 2. Standardized genetic (*A*), shared environmental (*C*), and non-shared environmental (*E*) variances of educational performance moderated by quintiles of school quality in a non-parametric gene-environment interaction analysis.

Note. Based on a model using a genetic correlation of rSS_G=.70, controlled for sex and year of birth.

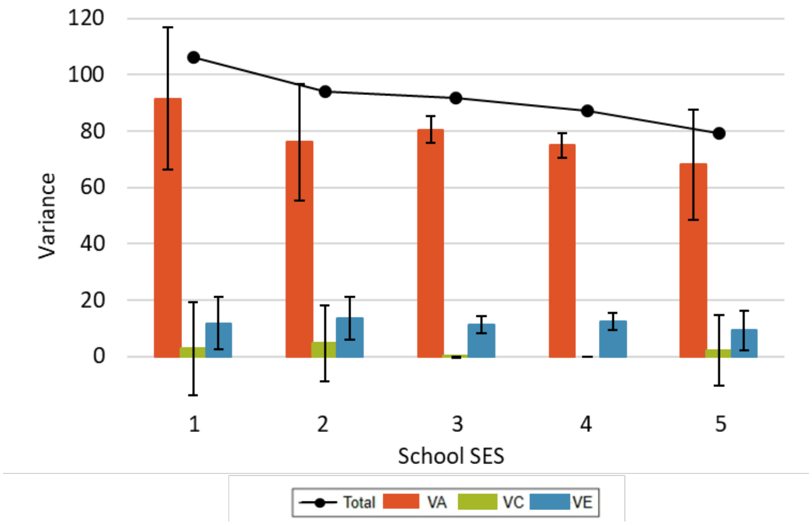

Supplementary Figure 3. Unstandardized genetic (*A*), shared environmental (*C*), and non-shared environmental (*E*) variances of educational performance moderated by quintiles of school SES in a non-parametric gene-environment interaction analysis, including 95% CI.

Note. Based on a model using a genetic correlation of rSS_G=.70, controlled for sex and year of birth.

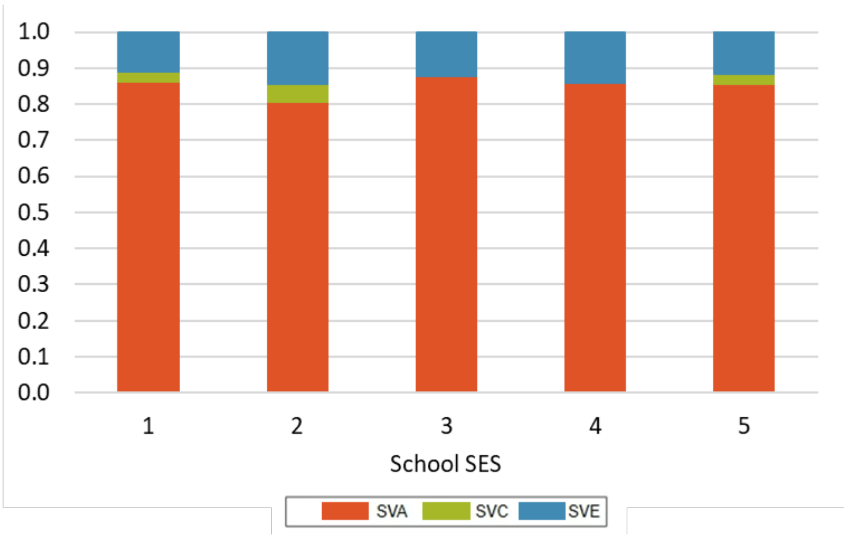

Supplementary Figure 4. Standardized genetic (*A*), shared environmental (*C*), and non-shared environmental (*E*) variances of educational performance moderated by quintiles of school SES in a non-parametric gene-environment interaction analysis.

Note. Based on a model using a genetic correlation of rSS_G=.70, controlled for sex and year of birth

Appendix C. Results school quality dimensions

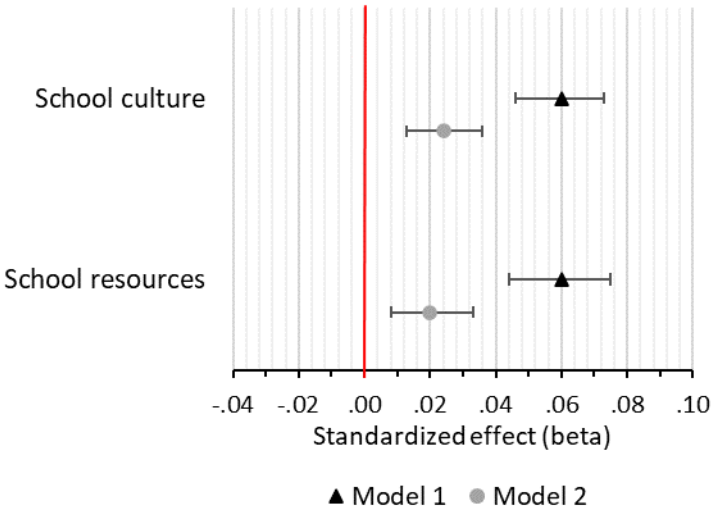

Supplementary Figure 5. Main effects of the school culture and school resources on educational performance, including 95% CI.

Note. Based on separate analyses for each school quality factor (Model 1), controlled for school SES and parental SES (Model 2). Both models control for sex and year of birth. N_{SSpairs} = 18,384; N_{OSpairs} = 11,050.

Supplementary Table 4. *ACE model for cito with main effects and moderation effects of school quality (school resources or culture), school SES, and parental SES ($N_{SSpairs}$ = 18,384, $N_{OSpairs}$ =11,050).*

	**SQ = school resources**				**SQ = school culture**			
	**Model 1**		**Model 2**		**Model 1**		**Model 2**	
**Parameter**	**Estimate**	**s.e.**	**Estimate**	**s.e.**	**Estimate**	**s.e.**	**Estimate**	**s.e.**
Intercept	534.63***	(0.08)	534.67***	(0.07)	534.63***	(0.08)	534.67***	(0.06)
a	9.28***	(0.06)	8.21***	(0.07)	9.28***	(0.06)	8.21***	(0.07)
$b_a SQ$	-0.20**	(0.06)	-0.01	(0.07)	-0.18**	(0.06)	0.00	(0.06)
$b_a SchoolSES$			-0.31***	(0.06)			-0.31***	(0.06)
$b_a ParentalSES$			-0.61***	(0.06)			-0.62	(0.06)
c	[a]		0.25	(0.19)	0.00	(0.03)	0.25	(0.19)
$b_c SQ$	[a]		-0.33	(0.27)	0.00	(0.01)	-0.29	(0.25)
$b_c SchoolSES$			-0.85***	(0.23)			-0.86***	(0.22)
$b_e ParentalSES$			0.72***	(0.19)			0.73***	(0.19)
e	2.98***	(0.12)	3.92***	(0.09)	2.98***	(0.12)	3.92***	(0.09)
$b_e SQ$	0.09***	(0.12)	-0.01	(0.08)	0.05	(0.12)	-0.05	(0.09)
$b_e SchoolSES$			-0.04	(0.09)			-0.04	(0.09)
$b_e ParentalSES$			-0.23	(0.08)			-0.22**	(0.08)
School quality	0.66***	(0.09)	0.21**	(0.07)	0.62***	(0.07)	0.23***	(0.06)
School SES			0.91**	(0.06)			0.91***	(0.06)
Parental SES			2.94	(0.05)			2.94***	(0.05)
Freely estimated parameters		11		21		13		21
Loglikelihood		-217438.01		-214782.43		-217429.35		-214778.46
Scaling correction factor		2.83		1.91		2.09		1.71
AIC		434898.01		429606.87		434884.69		429598.92

Note. * $p < .05$, ** $p < .01$, *** $p < .001$ (two-tailed test). [a] Fixed to zero for model convergence. A genetic correlation of $rSS_G = .70$ is used. Controlled for sex and year of birth. All continuous independent variables are z-standardized prior to the analyses. Robust standard errors accounting for clustering at the school level are shown in parentheses. Parameters *a*, *c*, and *e* refer to the unmoderated path coefficients capturing genetic, shared-environmental, and non-shared environmental influences, respectively. The *b* coefficients refer to the moderation effects of *a, c*, and *e*, by school quality (i.e., resources or culture), school SES, and parental SES.

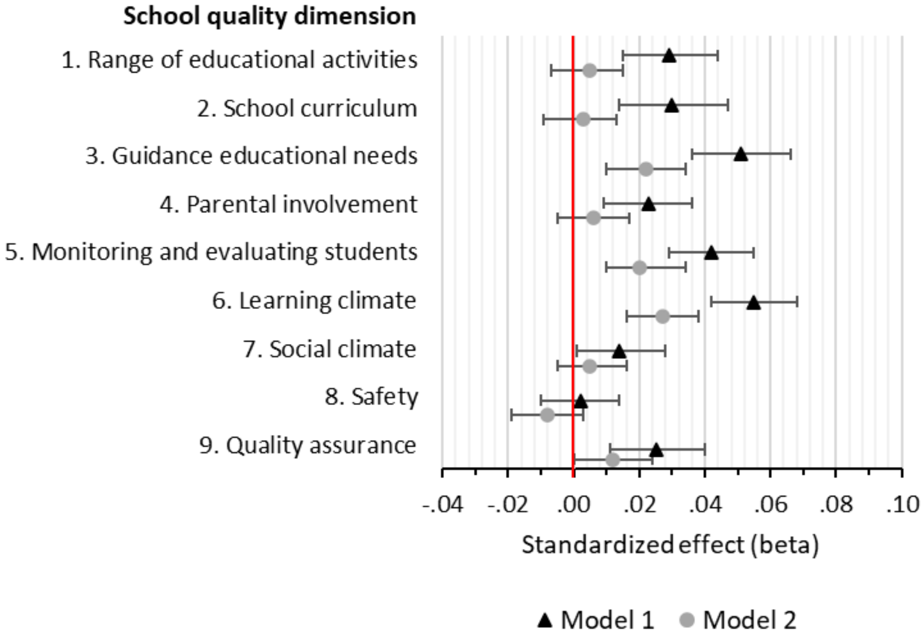

Supplementary Figure 6. Main effects of the school quality dimensions on educational performance, including 95% CI.

Note. Based on separate analyses for each school quality dimensions (Model 1), controlled for school SES and parental SES (Model 2). Both models control for sex and year of birth. $N_{SSpairs}$ = 18,384, $N_{OSpairs}$ =11,050.

Supplementary Table 5. *Moderation effects of selected school quality dimensions (Model 1), controlled for the moderation by school SES and parental SES (Model 2) (*$N_{SSpairs}$ *= 18,384,* $N_{OSpairs}$ *=11,050).*

	**3. Guidance of educational needs**				**5. Monitoring and evaluating**				**6. Learning climate**			
	**Model 1**		**Model 2**		**Model 1**		**Model 2**		**Model 1**		**Model 2**	
**Parameter**	**Estimate**	**s.e.**	**Estimate**	**s.e.**	**Estimate**	**s.e.**	**Estimate**	**s.e.**	**Estimate**	**s.e.**	**Estimate**	**s.e.**
Intercept	534.63***	(0.08)	534.67***	(0.06)	534.63***	(0.08)	534.67***	(0.07)	534.63***	(0.08)	534.67***	(0.06)
a	9.24***	(0.08)	8.22***	(0.07)	9.29***	(0.06)	8.22***	(0.07)	9.23***	(0.14)	8.19***	(0.07)
$b_a SQ$	-0.09	(0.14)	0.00	(0.07)	-0.15**	(0.05)	-0.05	(0.06)	-0.26	(0.14)	-0.10	(0.08)
$b_a SchoolSES$			-0.32***	(0.06)			-0.31***	(0.06)			-0.32***	(0.07)
$b_a ParentalSES$			-0.62***	(0.06)			-0.62***	(0.06)			-0.61***	(0.06)
c	-0.05	(0.96)	0.26	(0.18)	0.00	(0.04)	0.27	(0.19)	0.66	(1.01)	-0.22	(0.23)
$b_c SQ$	-0.78	(0.45)	-0.19	(0.21)	0.00	(0.00)	-0.09	(0.26)	0.46	(0.36)	0.65*	(0.27)
$b_c SchoolSES$			-0.86***	(0.22)			-0.87***	(0.22)			0.77**	(0.25)
$b_c ParentalSES$			0.72***	(0.19)			0.71***	(0.19)			-0.68**	(0.20)
e	3.02***	(0.13)	3.91***	(0.09)	2.96***	(0.12)	3.91***	(0.09)	3.02***	(0.17)	3.93***	(0.09)
$b_e SQ$	0.01	(0.18)	-0.01	(0.09)	0.07	(0.10)	0.00	(0.08)	0.15	(0.17)	0.01	(0.10)
$b_e SchoolSES$			-0.05	(0.09)			-0.05	(0.09)			-0.04	(0.09)
$b_e ParentalSES$			-0.23**	(0.08)			-0.23**	(0.08)			-0.23**	(0.08)
School quality	0.55***	(0.08)	0.22**	(0.07)	0.43***	(0.07)	0.18**	(0.06)	0.55***	(0.06)	0.24***	(0.05)
School SES			0.92***	(0.06)			0.92***	(0.06)			0.90***	(0.06)
Parental SES			2.94***	(0.05)			2.94***	(0.05)			2.94***	(0.05)
Freely estimated parameters		13		21		13		21		13		21
Loglikelihood		-217454.58		-214781.47		-217463.41		-214781.28		-217435.49		-214771.26
Scaling correction factor		2.26		1.72		2.22		1.80		2.76		2.08
AIC		434935.16		429604.94		434952.82		429604.55		434896.98		429584.52

Note. * $p < .05$, ** $p < .01$, *** $p < .001$ (two-tailed test). Controlled for sex and year of birth. A genetic correlation of rSS_G=.70 is used. All continuous independent variables are z-standardized prior to the analyses. Robust standard errors accounting for clustering at the school level are shown in parentheses. Parameters *a*, *c*, and *e* refer to unmoderated path coefficients capturing genetic, shared-environmental, and non-shared environmental influences, respectively. The *b* coefficients refer to the moderation effects of *a, c*, and *e*, by the school quality dimension, school SES, and parental SES.

Appendix D. Censoring

A potential problem with using *Cito* scores is that there may be less variance with increasing SES due to censoring. Therefore, we checked if the total variance in the raw test score also decreases with increasing SES. The raw test score consists of the total number of correctly answered test items and is normally distributed without censoring at the top. Hence, if we find decreasing variance of the raw scores too, we can be confident that the decreasing variance of the *Cito* score is not solely driven by ceiling effects. Even though censoring is no issue when using the raw scores, we do not use the raw score for our analyses. This is because the raw score is – in contrast to the *Cito* score – less comparable over the years. Moreover, we are especially interested in the *Cito* score because this is a meaningful measure in the Dutch contract given its importance for children's future educational career. Therefore, it is worthwhile to study this reality, including the associated properties of the scale.

We test for the influence of parental SES on the variance of the raw test score on the individual level with adjusted standard errors for clustering in families. The result is shown in Figure D1. We find a statistically significant association between parental SES and the total variance (b = -9.64, *robust s.e.* = 2.89, p = .001).

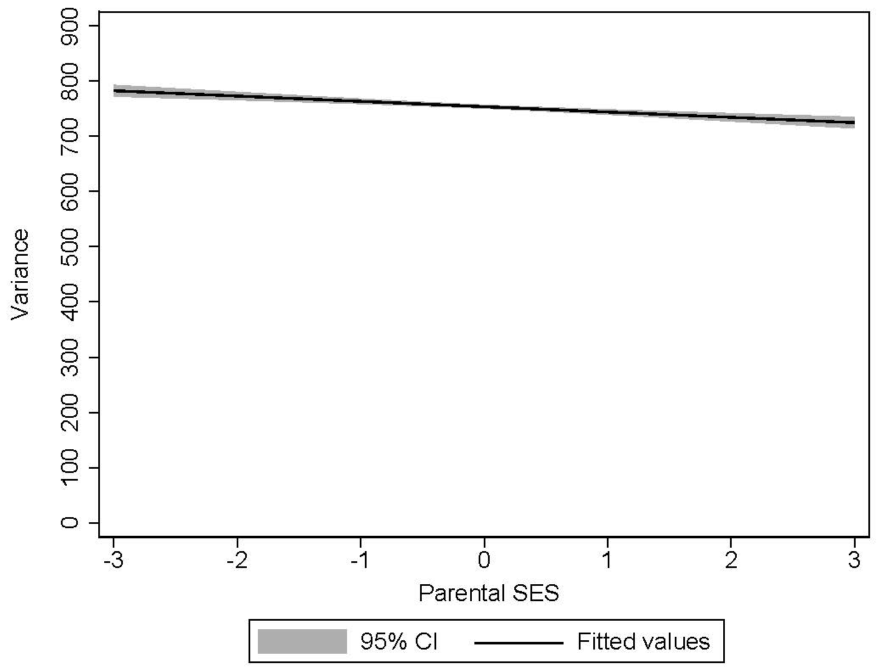

Supplementary Figure 7. Association between parental SES and raw test score variance.
Note. Including 95% CI, N = 52,096.

Appendix E. Measurement model school quality

Several steps were taken to construct this variable. First, we correlated all the items with the average Cito score of the school.[1] There were only a few items that correlated well with average educational performance, so we included also low positive correlations (larger than ~.030) that were statistically significant. Second, we divided the items into items that are related to school resources and to school climate. Third, we ran two Exploratory Factor Analyses, one for school resources and one for school climate. Several criteria were used for extracting factors: Kaiser's criterion (eigenvalue >1), inspection of the scree plot, and interpretability criteria. The interpretability criteria were: there should be at least two items with substantial loading (>.3), items that load on a factor should share conceptual meaning, and the rotated factor pattern should demonstrate a simple structure (no cross-loadings; items that load high on one factor have low loadings on the other factors). We extracted the resulting number of factors and excluded items that did not load (loading <.3) on any of the factors. All the resulting models have a good model fit (RMSEA, CFI, SRMR).[2]

This procedure led to two dimensions of school resources (Supplementary Table 6), and seven dimensions of school climate (Supplementary Table 7). We saved these nine dimensions as nine factor scores and constructed one overall factor out of them by using the nine factor scores in a new Confirmatory Factor Analysis (CFA). As an alternative operationalization, we excluded the dimensions that had a low loading on the overall school quality factor (Supplementary Table 8) and/or had a low correlation with schools' average Cito score (Supplementary Table 9). This alternative operationalization based on the remaining six dimensions did not lead to substantially different results and therefore we kept all the nine dimensions in.

To correct for measurement error, we calculated the error variance of the nine school quality indicators (that are saved as factor scores) and specified this in the measurement model when constructing the overall school quality factor. Also, we specified the error variance for the overall school quality factor when it was included as a single indicator variable in the analytical model. The error variance (see Brown, 2015) is calculated as

---

[1] We did this to optimize our measure for school quality. We also extracted the factor scores without dropping the items that did not correlate with school-level educational performance. The resulting school quality variable had a slightly lower main effect on students' educational performance, but it did not substantially change our results.

[2] We started with including data from the Inspectorate of Education (covering the school quality indicators) and a second data source, namely data from the education Executive Agency for the period 2011-2019 (covering administrative data, including school funding, number of pupils, number of staff). Following the steps that are described above, none of the items originating from this second data source ended up in our school quality factor.

$$Var(error) = Var(X) \times (1 - \rho_X),$$

where *Var(error)* is the error variance of the single indicator variable *X*, *Var(X)* is the total variance of the single indicator variable, and ρ_X is the reliability coefficient of the single indicator variable. The reliability coefficient is calculated by

$$\rho_X = \frac{VA \times Lsum^2}{VA \times Lsum^2 + Vsum + 2 \times Csum},$$

where *VA* is the variance of the factor, *Lsum* is the sum of all item loadings, *Vsum* is the sum of all residual variances of the items, and *Csum* is the sum of all residual covariances (which is zero if no covariance parameters are included in the measurement model, as in our case). We have two sets of single indicator variables. First, the nine school quality dimensions, and second, the two second-order overall school quality factors. The resulting reliability coefficients and error variances can be found in Supplementary Table 8.

Supplementary Table 6. *Factor analysis for the resources items ($N_{schools}$ = 9,709).*

**Label**	**Range of educational activities**	**(Implementation of) school curriculum**
The school provides a wide range of activities aimed at acquiring knowledge, insight, skills, and attitudes.	.86 (.01)	
The school ensures that there is coherence between the subjects concerning cross-curricular skills.	.50 (.02)	
General, cross-curricular skills and attitudes are part of the supply in the relevant subjects.	.87 (.02)	
The school ensures that the actual educational program corresponds to the planned program.		.47 (.05)
For groups of pupils, the school ensures a school curriculum that fits the educational needs of the pupils.		.45 (.05)
For individual pupils, the school ensures a school curriculum that fits the educational needs of the pupils.		.52 (.04)
The school curriculum is adjusted to the educational needs of the pupils		.48 (.04)
The school curriculum prepares pupils for further education.		.39 (.06)
The school curriculum is partly aimed at the desired language development of the pupils.		.37 (.03)
School varies the amount of time for education, depending on the educational needs of the pupils.		.36 (.04)

Note. All factor loadings are statistically significant (p <.001, two-tailed test). Standard error in parentheses. Standardized effects.

Supplementary Table 7. *Factor analysis for the climate items ($N_{schools}$ = 9,709).*

**Item**	**Guidance of educational needs**		**Parental involvement**		**Monitoring and evaluating (special needs) students**		**Learning climate**	**Social climate**	**Safety**	**Quality assurance**
The school ensures guidance for pupils with specific educational needs.	.95	(.01)								
The asks for help from pupils who drop out are analyzed by the school.	.40	(.02)								
The school takes care for additional guidance for pupils who require care.	1.02	(.01)								
The school has organized care and guidance in a structural way.	.33	(.02)								
The school has favorable conditions for the guidance of pupils with specific educational needs.	.33	(.02)								
The school involves parents/guardians in the guidance of the school career of the pupils.			.41	(.04)						
Parents are widely informed about the state of affairs at school.			.40	(.04)						
School contacts			.92	(.02)						
Contact with parents			1.01	(.02)						
The teachers systematically follow and analyze the progress in development of the pupils.					.49	(.02)				
The school identifies which pupils need care in an early stage.					.32	(.02)				
The school regularly evaluates the effects of the care.					.52	(.02)				
The school implements the care systematically.					.36	(.02)				

The school evaluates the results of the pupils yearly.	.46 (.02)				
The school ensures the quality of the learning process.	.46 (.02)				
The school analyses the nature of the care that special need pupils require, based on the collected data.	.50 (.02)				
The teachers explain clearly.		.31 (.02)			
The teachers create a task-oriented work environment.		.67 (.03)			
The pupils are active and engaged.		.60 (.03)			
Staff and pupils interact positively.			.33 (.08)		
The school takes care of a pleasant, motivating environment for pupils.			.91 (.09)		
The atmosphere at school is stimulating.			.83 (.09)		
The school has a safety policy.				.53 (.06)	
The school ensures safety.				1.00 (.09)	
The involvement of staff, parents, students, and external parties in the self-evaluation is guaranteed.					.66 (.02)
The school has procedures, plans, and instruments for a self-evaluation.					.73 (.02)
School is accountable to stakeholders about the achieved educational quality.					.38 (.02)

Note. All factor loadings are statistically significant (p <.001, two-tailed test). Standard error in parentheses. Standardized effects.

Supplementary Table 8. *Factor loadings, estimated reliability, and error variance for the dimensions measuring school quality ($N_{schools}$ = 9,709).*

	ρ	Error variance	SQ1		SQ2	
1. Range of educational activities	.82	.14	.61	(.02)	.53	(.02)
2. (Implementation of) school curriculum	.65	.02	.38	(.02)	.30	(.02)
3. Guidance of educational needs	.79	.19	.63	(.02)	.56	(.02)
4. Parental involvement	.91	.02	.34	(.02)	[a]	
5. Monitoring and evaluating (special needs) students	.61	.08	.58	(.02)	.70	(.02)
6. Learning climate	.55	.05	.55	(.02)	.66	(.02)
7. Social climate	.79	.04	.25	(.03)	[a]	
8. Safety	.77	.07	.26	(.03)	[a]	
9. Quality assurance	.65	.18	.66	(.01)	.62	(.02)
ρ			.72		.74	
Error variance			.09		.06	

Note. All factor loadings are statistically significant (p <.001, two-tailed test). Standard error in parentheses. Standardized effects. SQ = school quality.

[a] Excluded because of low factor loading and/or low correlation with average cito score.

Supplementary Table 9. *Correlations between the dimensions and factor scores for school quality ($N_{schools}$ = 9,709) and average cito score of the school ($N_{schools}$ = 7,622).*

	1.	2.	3.	4.	5.	6.	7.	8.	9.	10.	11.
1. Range of educational activities											
2. (Implementation of) school curriculum	.27***										
3. Guidance of educational needs	.40***	.33***									
4. Parental involvement	.18***	.18***	.21***								
5. Monitoring and evaluating (special needs) students	.19***	.10***	.26***	.10***							
6. Learning climate	.27***	.11***	.27***	.09***	.58***						
7. Social climate	.16***	.16***	.11***	.19***	.04***	.02					
8. Safety	.14***	.19***	.07***	.20***	.07***	.06***	.48***				
9. Quality assurance	.37***	.18***	.30***	.25***	.41***	.27***	.12***	.12***			
10. Factor score school quality 1 (9 dimensions)	.66***	.44***	.66***	.39***	.65***	.62***	.28***	.29***	.70***		
11. Factor score school quality 2 (6 dimensions)	.57***	.35***	.59***	.23***	.78***	.75***	.12***	.14***	.66***	.96***	
12. Cito (school)	.07***	.09***	.10***	.05***	.14***	.14***	.04***	.05***	.10***	.16***	.17***

Note. *** p <.001 (two-tailed test).

Supplementary Table 10. *Factor loadings for the resources and climate dimensions of school quality ($N_{schools}$ = 9,709).*

	**Resources**		**Climate**	
1. Range of educational activities	.71	(.02)		
2. (Implementation of) school curriculum	.42	(.02)		
3. Guidance of educational needs			.61	(.02)
4. Parental involvement			.33	(.02)
5. Monitoring and evaluating (special needs) students			.61	(.03)
6. Learning climate			.57	(.03)
7. Social climate			.23	(.03)
8. Safety			.25	(.03)
9. Quality assurance			.67	(.01)
ρ	.48		.66	
Error variance	.22		.10	

Note. All factor loadings are statistically significant (p <.001, two-tailed test). Standard error in parentheses. Standardized effects.

Appendix F. Measurement model socioeconomic status (SES)

Supplementary Table 11. *Descriptive statistics and factor loadings for socioeconomic status.*

	N	**Mean**	**S.D.**	**SES**	
ISCED father	1,023,068	4.32	2.11	.68	(<.01)
ISCED mother	1,148,977	3.95	2.08	.80	(<.01)
Income (percentile) father	1,749,550	75.93	22.54	.43	(<.01)
Income (percentile) mother	1,620,932	44.29	25.86	.51	(<.01)

Note. All factor loadings are statistically significant (p <.001, two-tailed test). Standard error in parentheses. All items were standardized prior to the factor analysis.

Appendix G. Intraclass Correlation Coefficients

We compare the ICC of SS and OS twins with SS and OS siblings. Sibling pairs were selected by choosing a random sibling in a family with a co-sibling who is closest in age. In order to increase the comparability of sibling pairs with twin pairs, we only included closely spaced siblings with a maximum age difference of three years. In addition, we made the same sample selections as for twins: birth cohorts 1994-2007, available cito scores, attending the same primary school, and excluding missings on school quality.

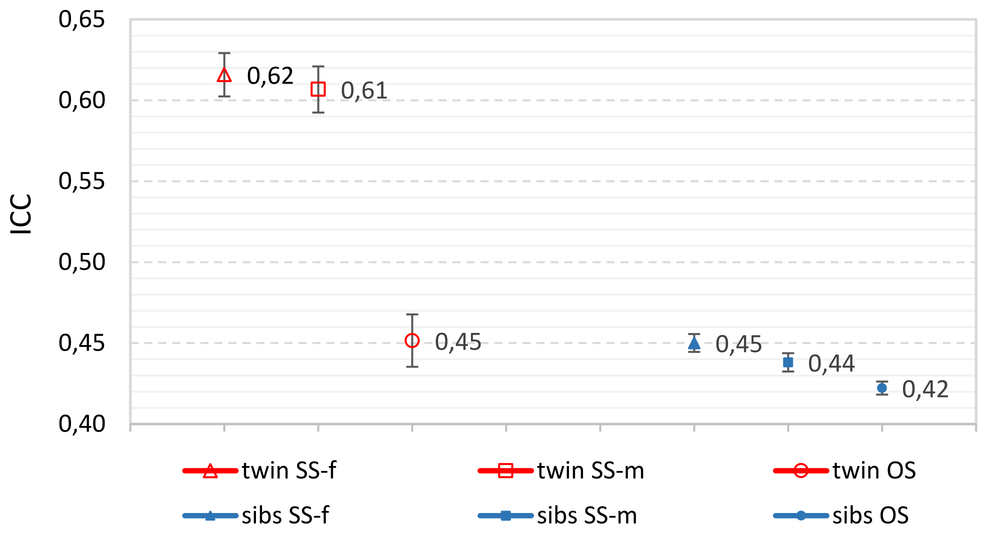

Supplementary Figure 8. Intraclass correlation coefficient (ICC) for cito-scores of same-sex female (SS-f), same-sex male (SS-m) and opposite-sex (OS) twin and sibling pairs., including 95% CI.
