# Supplementary material for: Gene-environment interaction analysis of school quality and educational inequality
Source: NPJ Sci Learn. 2024 Mar 1;9:14. doi: 10.1038/s41539-024-00225-x (PMC10907386; doi:10.1038/s41539-024-00225-x)
Supplement: Supplementary file 2 — Reporting summary [file 41539_2024_225_MOESM2_ESM.pdf]

nature portfolio

Corresponding author(s): Kim Stienstra
Last updated by author(s): Oct 31, 2023

Reporting Summary

Nature Portfolio wishes to improve the reproducibility of the work that we publish. This form provides structure for consistency and transparency in reporting. For further information on Nature Portfolio policies, see our Editorial Policies and the Editorial Policy Checklist.

Statistics

For all statistical analyses, confirm that the following items are present in the figure legend, table legend, main text, or Methods section.

n/a	Confirmed	
☐	☒	The exact sample size (*n*) for each experimental group/condition, given as a discrete number and unit of measurement
☐	☒	A statement on whether measurements were taken from distinct samples or whether the same sample was measured repeatedly
☐	☒	The statistical test(s) used AND whether they are one- or two-sided<br>*Only common tests should be described solely by name; describe more complex techniques in the Methods section.*
☐	☒	A description of all covariates tested
☐	☒	A description of any assumptions or corrections, such as tests of normality and adjustment for multiple comparisons
☐	☒	A full description of the statistical parameters including central tendency (e.g. means) or other basic estimates (e.g. regression coefficient) AND variation (e.g. standard deviation) or associated estimates of uncertainty (e.g. confidence intervals)
☐	☒	For null hypothesis testing, the test statistic (e.g. *F*, *t*, *r*) with confidence intervals, effect sizes, degrees of freedom and *P* value noted<br>*Give P values as exact values whenever suitable.*
☒	☐	For Bayesian analysis, information on the choice of priors and Markov chain Monte Carlo settings
☐	☒	For hierarchical and complex designs, identification of the appropriate level for tests and full reporting of outcomes
☒	☐	Estimates of effect sizes (e.g. Cohen's *d*, Pearson's *r*), indicating how they were calculated

Our web collection on statistics for biologists contains articles on many of the points above.

Software and code

Policy information about availability of computer code

Data collection	No software was used
Data analysis	Code is available at https://osf.io/xsgdt/

For manuscripts utilizing custom algorithms or software that are central to the research but not yet described in published literature, software must be made available to editors and reviewers. We strongly encourage code deposition in a community repository (e.g. GitHub). See the Nature Portfolio guidelines for submitting code & software for further information.

Data

Policy information about availability of data

All manuscripts must include a data availability statement. This statement should provide the following information, where applicable:

- Accession codes, unique identifiers, or web links for publicly available datasets
- A description of any restrictions on data availability
- For clinical datasets or third party data, please ensure that the statement adheres to our policy

All results are based on own calculations using non-public microdata from Statistics Netherlands. Under certain conditions, these microdata are accessible for statistical and scientific research. For further information, see https://www.cbs.nl/en-gb/onze-diensten/customised-services-microdata/microdata-conducting-your-own-research or e-mail microdata@cbs.nl.

Research involving human participants, their data, or biological material

Policy information about studies with human participants or human data. See also policy information about sex, gender (identity/presentation), and sexual orientation and race, ethnicity and racism.

Reporting on sex and gender	Our design relies on comparison between same-sex and opposite-sex twin pairs. Sex is based on official registration in the municipal population registers.
Reporting on race, ethnicity, or other socially relevant groupings	We use a continuous measure of socioeconomic status, a construct created based on both parents income and level of education from administrative registers. We use the percentile scores of personal yearly income, which includes the gross income from labor, own company, income insurance benefits, and social security benefits (excluding child benefits and child-related budget). Premiums for income insurance have been deducted. For education, we rely on the highest attained level of education, which is coded according to the International Standard Classification of Education (ISCED) 2011.
Population characteristics	Children in the final grade of Dutch primary education (~age 12), birth cohorts 1994-2007.
Recruitment	As administrative data is used, recruitment or self-selection bias is not applicable.
Ethics oversight	This research was approved by the Ethics Committee of the Faculty of Social and Behavioral Sciences, Utrecht University (FETC20-216).

Note that full information on the approval of the study protocol must also be provided in the manuscript.

Field-specific reporting

Please select the one below that is the best fit for your research. If you are not sure, read the appropriate sections before making your selection.

☐ Life sciences ☒ Behavioural & social sciences ☐ Ecological, evolutionary & environmental sciences

For a reference copy of the document with all sections, see nature.com/documents/nr-reporting-summary-flat.pdf

Behavioural & social sciences study design

All studies must disclose on these points even when the disclosure is negative.

Study description	A classical twin study with unknown zygosity using quantitative data on educational performance
Research sample	Twins in the final grade of Dutch primary education (~age 12), birth cohorts 1994-2007.
Sampling strategy	Given the use of administrative data, sampling procedure and sample-size calculation is not applicable. We use all available data of our research sample described above.
Data collection	Diverse registers (including municipal personal records, education registers, tax registers) available via Statistics Netherlands
Timing	Not applicable
Data exclusions	Data excluded from twins<br>- when both twins of a pair had no cito data available (Npairs 16,570)<br>- who attended different schools (Npairs 563)<br>- of whom the school is unkown (Npairs 184)<br>- of whom no school information is available (N pairs 196)<br>- of whom no family SES data is available (Npairs 38)<br><br>Missings on schools and families had to be excluded, because they cannot be handled in ACE models with continuous moderation.
Non-participation	Not applicable
Randomization	Not applicable

Reporting for specific materials, systems and methods

We require information from authors about some types of materials, experimental systems and methods used in many studies. Here, indicate whether each material, system or method listed is relevant to your study. If you are not sure if a list item applies to your research, read the appropriate section before selecting a response.

Materials & experimental systems

n/a	Involved in the study
☒	☐ Antibodies
☒	☐ Eukaryotic cell lines
☒	☐ Palaeontology and archaeology
☒	☐ Animals and other organisms
☒	☐ Clinical data
☒	☐ Dual use research of concern
☒	☐ Plants

Methods

n/a	Involved in the study
☒	☐ ChIP-seq
☒	☐ Flow cytometry
☒	☐ MRI-based neuroimaging

Plants

Seed stocks

Report on the source of all seed stocks or other plant material used. If applicable, state the seed stock centre and catalogue number. If plant specimens were collected from the field, describe the collection location, date and sampling procedures.

Novel plant genotypes

Describe the methods by which all novel plant genotypes were produced. This includes those generated by transgenic approaches, gene editing, chemical/radiation-based mutagenesis and hybridization. For transgenic lines, describe the transformation method, the number of independent lines analyzed and the generation upon which experiments were performed. For gene-edited lines, describe the editor used, the endogenous sequence targeted for editing, the targeting guide RNA sequence (if applicable) and how the editor was applied.

Authentication

Describe any authentication procedures for each seed stock used or novel genotype generated. Describe any experiments used to assess the effect of a mutation and, where applicable, how potential secondary effects (e.g. second site T-DNA insertions, mosiacism, off-target gene editing) were examined.
